# Opioid Drug-Drug-Drug Interactions and Unintentional Traumatic Injury: Screening to Detect Three-Way Drug Interaction Signals

**DOI:** 10.3389/fphar.2022.845485

**Published:** 2022-05-10

**Authors:** Emily K. Acton, Sean Hennessy, Colleen M. Brensinger, Warren B. Bilker, Todd A. Miano, Sascha Dublin, John R. Horn, Sophie Chung, Douglas J. Wiebe, Allison W. Willis, Charles E. Leonard

**Affiliations:** ^1^ Center for Pharmacoepidemiology Research and Training, Center for Clinical Epidemiology and Biostatistics, Perelman School of Medicine, University of Pennsylvania, Philadelphia, PA, United States; ^2^ Department of Biostatistics, Epidemiology, and Informatics, Perelman School of Medicine, University of Pennsylvania, Philadelphia, PA, United States; ^3^ Translational Center of Excellence for Neuroepidemiology and Neurology Outcomes Research, Department of Neurology, Perelman School of Medicine, University of Pennsylvania, Philadelphia, PA, United States; ^4^ Leonard Davis Institute of Health Economics, University of Pennsylvania, Philadelphia, PA, United States; ^5^ Department of Systems Pharmacology and Translational Therapeutics, Perelman School of Medicine, University of Pennsylvania, Philadelphia, PA, United States; ^6^ Department of Psychiatry, Perelman School of Medicine, University of Pennsylvania, Philadelphia, PA, United States; ^7^ Kaiser Permanente Washington Health Research Institute, Kaiser Permanente Washington, Seattle, WA, United States; ^8^ Department of Epidemiology, School of Public Health, University of Washington, Seattle, WA, United States; ^9^ Department of Pharmacy, School of Pharmacy, University of Washington, Seattle, WA, United States; ^10^ AthenaHealth, Inc., Watertown, MA, United States; ^11^ Penn Injury Science Center, University of Pennsylvania, Philadelphia, PA, United States

**Keywords:** drug interactions, injury, opioid analgesics, pharmacoepidemiology, population health, self-controlled case series

## Abstract

Growing evidence suggests that drug interactions may be responsible for much of the known association between opioid use and unintentional traumatic injury. While prior research has focused on pairwise drug interactions, the role of higher-order (i.e., drug-drug-drug) interactions (3DIs) has not been examined. We aimed to identify signals of opioid 3DIs with commonly co-dispensed medications leading to unintentional traumatic injury, using semi-automated high-throughput screening of US commercial health insurance data. We conducted bi-directional, self-controlled case series studies using 2000–2015 Optum Data Mart database. Rates of unintentional traumatic injury were examined in individuals dispensed opioid-precipitant base pairs during time exposed vs unexposed to a candidate interacting precipitant. Underlying cohorts consisted of 16–90-year-olds with new use of opioid-precipitant base pairs and ≥1 injury during observation periods. We used conditional Poisson regression to estimate rate ratios adjusted for time-varying confounders, and semi-Bayes shrinkage to address multiple estimation. For hydrocodone, tramadol, and oxycodone (the most commonly used opioids), we examined 16,024, 8185, and 9330 drug triplets, respectively. Among these, 75 (0.5%; hydrocodone), 57 (0.7%; tramadol), and 42 (0.5%; oxycodone) were significantly positively associated with unintentional traumatic injury (50 unique base precipitants, 34 unique candidate precipitants) and therefore deemed potential 3DI signals. The signals found in this study provide valuable foundations for future research into opioid 3DIs, generating hypotheses to motivate crucially needed etiologic investigations. Further, this study applies a novel approach for 3DI signal detection using pharmacoepidemiologic screening of health insurance data, which could have broad applicability across drug classes and databases.

## 1 Introduction

Opioid-related morbidity and mortality are pressing public health challenges in the US, with more than 197,000 emergency department visits, 91,000 hospitalizations, and 42,000 deaths associated with opioids in 2016 alone (Centers for Disease Control and Prevention, 2018; Centers for Disease Control and Prevention, 2019). There is a growing body of evidence demonstrating that major drivers of this morbidity and mortality are unintentional traumatic injuries (i.e., fractures, motor vehicle accidents, etc.) resulting from the central nervous system (CNS) depressant effects of opioids (Buckeridge et al., 2010; Yoshikawa et al., 2020; Cameron-Burr et al., 2021). Pharmacodynamic and pharmacokinetic opioid-drug interactions can significantly potentiate these opioid CNS depressant effects, and thus represent key contributors to opioid-induced traumatic injuries (Gudin, 2012; Bain and Knowlton, 2019; Matos et al., 2020). Because detecting such interactions could play a critical role in preventing opioid-related injuries, we previously conducted a large-scale screening of real-world data to identify signals of injury-inducing opioid interactions with commonly co-prescribed drugs (Leonard et al., 2020). However, an intrinsic limitation of our prior screening study, and investigations into opioid interactions more generally, is the exclusive focus on pairwise interactions that fails to identify higher-order interactions.

Despite considerable attention being focused on drug interactions as a significant source of preventable iatrogenic harm, higher-order interactions, such as drug-drug-drug interactions (3DIs), remain underappreciated and understudied (Horn and Hansten, 2011). This knowledge gap has become particularly noteworthy in the context of rising polypharmacy rates worldwide (Wastesson et al., 2018; World Health Organization, 2019), which continue to increase the probability and relevance of 3DIs (Létinier et al., 2021). While some 3DIs may be postulated based purely on knowledge of pairwise interactions, real-world evidence is often needed to determine the clinical effects on patient outcomes from multiple coincident pharmacokinetic and/or pharmacodynamic drug interactions (Horn and Hansten, 2011). These assessments of 3DIs are especially important for medications that are frequently received by populations with high rates of polypharmacy, such as among those populations for which opioids are often indicated. Estimates suggest that people who use opioids fill a mean of 52 prescriptions annually from approximately 10 drug classes, and that opioids represent the most common component of polypharmacy among CNS-active medications (Medicare Payment Advisory Committee, 2015; Gerlach et al., 2017; Matos et al., 2020). While these findings illustrate the critical need for research on higher-order interactions, the lack of established strategies for assessing 3DIs has stymied such investigations.

Therefore, we sought to accomplish the following objectives: 1) to develop and implement a semi-automated, high-throughput approach to screen for 3DIs using pharmacoepidemiologic methods applied to administrative healthcare databases; and 2) to identify signals of opioid 3DIs with commonly co-dispensed medications associated with unintentional traumatic injury.

## 2 Materials and Methods

We conducted semi-automated, high-throughput pharmacoepidemiologic screening of Optum’s de-identified Clinformatics^®^ Data Mart Database (Optum Inc, 2014) administrative data from 5/1/2000-9/30/2015 (see Supplementary Methods for additional details on data source). From this large US commercial health insurance database, we sought to identify 3DI signals between opioid object + precipitant base pairs and candidate interacting precipitants. In the context of drug interactions, the object is the drug being affected (i.e., “victim”), whereas the precipitants are the drugs doing the affecting (i.e., “perpetrators”) (Hennessy et al., 2016). We first identified both the potential base pair precipitants and candidate interacting precipitants, which we operationalized as any orally administered, non-opioid drugs that were frequently co-dispensed with opioids. We constructed separate study cohorts for 16–90-year-old, new users of each combination of an opioid with a base pair precipitant, and identified 3DI signals by performing thousands of confounder-adjusted self-controlled case series studies. The goal of these studies was to examine the associations between different sets of opioid object + precipitant base pairs with candidate precipitants and the following outcomes: unintentional traumatic injury (primary outcome); typical hip fracture (secondary outcome); and motor vehicle crash while the person was driving (secondary outcome) (Supplementary Table S1).

**FIGURE 1:**
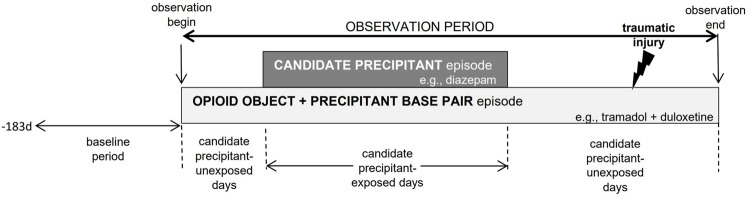
Example of opioid object + precipitant base pair exposure episode eligible for inclusion. The presence of candidate precipitant-unexposed person-days before and after candidate precipitant-exposed person-days is indicative of a bi-directional implementation of the self-controlled case series design.

For each drug triplet consisting of an opioid object-precipitant-precipitant set, we conducted a bi-directional self-controlled case series study to examine the rate of each outcome in an individual treated with the opioid object + precipitant base pairs during time exposed vs unexposed to the candidate precipitant. Figure 1 provides a graphical representation of the design. Although the “case series” phrase within self-controlled case series may seem to imply the absence of a comparator, the approach is a rigorous controlled self-matched epidemiologic study design; it is the cohort analogue of the case-crossover design (Maclure et al., 2012). The self-controlled case series design is ideal for drug interaction screening because: 1) the causal contrast is made *within* an individual and thus inherently controls for confounding by measured and unmeasured factors that remain constant within an individual over the observation period (e.g., sex, genetics); 2) the underlying statistical model can control for time-varying factors; (Lee and Carlin, 2014); 3) the approach is highly computationally-efficient (Whitaker et al., 2009), since it includes only persons experiencing an outcome; and 4) there is precedent for the use of high-throughput applications. Analogous pharmacoepidemiologic screening studies have identified drugs associated with hypoglycemia in people using insulin secretagogues (Han et al., 2017), rhabdomyolysis in people using statins (Bykov et al., 2019), and serious bleeding in people using clopidogrel (Leonard et al., 2019) and anticoagulants (Pottegård et al., 2014; Martín-Pérez et al., 2018; Bykov et al., 2019).

The University of Pennsylvania’s institutional review board approved this research under protocols #831486 and #819924.

### 2.1 Creating Study Cohorts of Persons With New Use of Opioid + Precipitant Base Pairs

Our study cohorts were composed of 16–90-year-olds, with this range selected to maximize capture of opioid 3DI signals across the age spectrum. While it may be reasonable to suspect greater risks of opioid 3DI-related traumatic injuries in older populations (i.e., due to greater numbers of co-prescribed drugs and baseline outcome risks, etc.), opioid dispensing rates remain high in younger US populations (Renny et al., 2021). Thus, identification of potentially clinically impactful 3DIs has real-world relevance across these age groups. The specific inclusion of persons ≥16 years of age also served to facilitate more complete assessment of our secondary outcome, motor vehicle crash while the person was driving (i.e., 16 is the youngest full-license legal driving age in the US).

We constructed separate study cohorts for new users of each combination of an opioid object drug with a base pair precipitant. We achieved this by requiring a baseline period (see “Defining observation and baseline periods”) without dispensing for the given opioid in the base pair. We then utilized pharmacy claim dates and days’ supply values to build opioid object + precipitant base pair exposure episodes consisting of ≥1 dispensings of the opioid object and base pair precipitant. We permitted a grace period between contiguous dispensings and at the end of the last dispensing for the opioid objects and base pair precipitants. The length of the grace period was calculated as days’ supply × 0.20.

See Supplementary Methods for additional details on identification of base pair precipitant drugs and candidate interacting precipitant drugs during opioid object drug use.

### 2.2 Defining Observation and Baseline Periods

For each new user of a base pair meeting inclusion criteria, we began the observation period at the start of concomitant use of the opioid object and base pair precipitant, and censored the observation period upon the earliest occurrence of: 1) lapsed exposure to the opioid object and/or base pair precipitant (permitting the grace period); 2) a switch from the opioid object to a pharmacologic alternative (i.e., a different opioid or opioid agonist-antagonist); 3) health plan disenrollment (allowing gaps of 45 days); or 4) the end of the study dataset. If inclusion criteria were met at more than one time during the study period, a single individual could have more than one observation period as part of different base pair assessments. Since the self-controlled case series design is a case-only approach, we required new users of base pairs to experience an outcome (see “Identifying outcomes”) during their observation period. We did not censor upon outcome occurrence since this would violate an underlying assumption of the self-controlled case series design (Whitaker et al., 2006; 2009).

We defined the baseline period as the 183 days immediately preceding yet excluding the first day of the observation period. We required the baseline period be devoid of an interruption in health plan coverage (allowing a maximum gap of 45 days) or dispensing for the opioid object drug under study. We did not exclude episodes preceded by a baseline dispensing for a pharmacologic alternative to the opioid object drug; this permitted us to study second- and later-line opioid therapies. For example, we excluded from the hydrocodone cohort episodes with baseline hydrocodone dispensing, but not episodes with baseline tramadol dispensing. We also did not exclude episodes with baseline dispensing of base pair precipitants, as it was infeasible to individually assess therapeutic alternatives for all base pair precipitants. We further required the 30 days immediately preceding and including the observation period start date be event-free; this served to minimize reverse causation since opioids are used to treat injury-induced pain.

### 2.3 Categorizing Observation Period Time Based on Candidate Precipitant Drug Exposure

The exposure of interest was use of the candidate interacting precipitant drug. We classified each day in the observation period either as exposed to the candidate precipitant or as unexposed to the candidate precipitant. Candidate precipitant exposed days were defined by exposure to the candidate interacting precipitant drug concomitant with the opioid object + precipitant base pair. Candidate precipitant unexposed days were all other observation period days (i.e., days with exposure to the opioid object + precipitant base pair, but no exposure to the candidate precipitant). To minimize exposure trend bias (Maclure et al., 2012), we included candidate precipitant-unexposed days occurring both before and after candidate precipitant-exposed days (see Supplementary Methods).

### 2.4 Defining the Covariates of Interest

The self-controlled case series design implicitly controls for time-invariant, but not time-varying, covariates (Whitaker et al., 2006). We therefore included in each regression model the following time-varying covariates assessed during each day of observation time: 1) opioid daily dose, in morphine milligram equivalents (see Supplementary Methods); (Centers for Medicare and Medicaid Services, 2018); and 2) presence of prior traumatic injury of interest in any diagnostic position on any claim type (e.g., a prior secondary-position ambulatory care hip fracture diagnosis in an analysis of the hip fracture endpoint). The latter covariate is relevant because prior injury may predict subsequent injury (Fulton et al., 2014), and the self-controlled case series design does not censor observation time upon event occurrence. In a secondary analysis, we further evaluated the impacts of prior injuries on our findings by removing episodes with unintentional traumatic injury prior to the first day of observation.

### 2.5 Identifying Outcomes

The primary outcome was unintentional traumatic injury, defined as an emergency department or inpatient hospitalization for fracture, dislocation, sprain/strain, intracranial injury, internal injury of thorax, abdomen, or pelvis, open wound, injury to blood vessels, crushing injury, injury to nerves or spinal cord, or certain traumatic complications and unspecified injuries. Consistent with the American College of Surgeons’ National Trauma Data Standard (American College of Surgeons, 2016), our definition excluded: 1) late effects of injuries, poisonings, toxic effects, and other external causes; 2) superficial injury; 3) contusion with intact skin surface; and 4) effects of a foreign body entering through an orifice. Consistent with work by (Sears et al., 2015), our definition also excluded burns, as such injuries are unlikely to be due to opioid use. A secondary outcome was inpatient hospitalization for typical hip fracture. We excluded: 1) pathologic hip fractures, since these events are due to a localized process such as malignancy or infection (Curtis et al., 2009); and 2) atypical hip fractures, since these events are infrequently traumatic and often attributed to bisphosphonate and/or glucocorticoid use (Shane et al., 2014). Another secondary outcome was motor vehicle crash while the person was driving, defined as an unintentional traumatic injury (see primary outcome above) plus an external cause of injury code for an unintentional traffic or nontraffic accident. We excluded crashes of a self-inflicted, assault, or undetermined manner, consistent with the Centers for Disease Control and Prevention’s injury mortality framework (Recommended framework for presenting injury mortality data, 1997). We provide operational outcome definitions, their operating characteristics, and other support for their use in Supplementary Table S1.

### 2.6 Statistical Analysis

For assessing each opioid object + precipitant base pair relative to each outcome, we constructed an analytic file in which the unit of observation was the person-day covered by active prescriptions for the opioid object and base pair precipitant. The binary dependent variable was whether the unintentional traumatic injury occurred on that day. Independent variables included a unique subject identifier, whether a person-day was exposed vs unexposed to the candidate precipitant, and the time-varying covariates (see “Defining the covariates of interest”). The parameter of interest was the outcome occurrence rate ratio (RR) during candidate precipitant-exposed vs candidate precipitant-unexposed days (i.e., rate exposed to opioid object + precipitant base pair and candidate precipitant/rate exposed to opioid object + precipitant base pair). We used conditional Poisson regression models (*xtpoisson* with *fe* option, Stata v.16: College Station, TX, United States) to estimate RRs and 95% confidence intervals (CIs) (Whitaker, 2005; Whitaker et al., 2006, 2009). To avoid statistically unstable estimates, we did not estimate RRs when there were: 1) < 5 candidate precipitant-exposed persons; or 2) no events during candidate precipitant-exposed time. Further, we do not report RRs from non-converged conditional Poisson regression models or if the variance of the beta estimate for the parameter of interest was >10.

To address multiple estimation inherent in calculating numerous RRs, we used a semi-Bayes shrinkage method. This approach improves the validity of effect estimates and preserves nominal type-1 error (Greenland and Poole, 1994; Steenland et al., 2000). See details in Supplementary Methods.

To contextualize findings, we compared 3DI signals generated by our semi-automated approach to putative, pairwise interactions documented in the following drug interaction knowledgebases: Micromedex (IBM Watson Health: Cambridge, MA, United States); and Facts & Comparisons Clinical Drug Information (Wolters Kluwer: Alphen aan den Rijn, South Holland, Netherlands).

## 3 Results

The three most commonly used opioids were hydrocodone, tramadol, and oxycodone, which are the focus of the presented results unless otherwise specified. Table 1 summarizes characteristics of persons experiencing traumatic injury while receiving opioid object drugs. There were 25,019, 12,650, and 10,826 people with new use of hydrocodone, tramadol, and oxycodone respectively, all of whom by design experienced an outcome. The three most commonly occurring injury types were sprain/strain (39.9%), certain traumatic complications and unspecified injuries (27.1%), and fracture (25.7%). Median durations of observation for hydrocodone, tramadol, and oxycodone were 12, 13, and 19 days, respectively. The plurality of hydrocodone (40.6%), tramadol (47.3%), and oxycodone (40.3%) users were Caucasian adult females. Few people using hydrocodone (18.1%), tramadol (17.3%), and oxycodone (15.0%) had multiple unintentional traumatic injuries during observation time. In analyses of secondary outcomes, cohorts consisted of 1,142, 848, and 461, and 246, 98, and 113 users for typical hip fracture and motor vehicle crash, respectively. Supplementary Tables S2, S3 summarize characteristics of persons experiencing these outcomes while receiving opioid object drugs.

**TABLE 1:** Descriptors of persons experiencing unintentional traumatic injury while receiving opioid object drugs.

		Object Drug											
		Codeine	Fentanyl	Hydrocod	Hydromor	Levorph	Meperidine	Methadone	Morphine	Oxycodone	Oxymorph	Tapentadol	Tramadol
Persons		6,065	1,435	25,019	828	2	248	439	1,612	10,826	224	163	12,650
Days of observation period, median (Q1-Q3) per person		10.0 (6.0–19.0)	33.0 (16.0–57.0)	12.0 (7.0–27.0)	13.0 (7.0–31.0)	26.5 (19.0–34.0)	9.0 (5.0–13.0)	50.0 (24.0–145.0)	35.0 (16.0–72.0)	13.0 (7.0–37.0)	38.5 (22.0–152.5)	24.5 (10.5–37.0)	19.0 (10.0–37.0)
Days of observation, sum		130,951	95,350	1,066,507	32,251	53	7,656	52,699	152,303	641,103	28,640	10,075	574,007
Unintentional traumatic injuries, sum		7,790	1,894	33,567	1,023	2	293	644	2,082	14,071	299	284	17,385
*Demographics*													
Age, median (Q1–Q3), years		54.5 (40.5–72.6)	73.8 (60.0–80.2)	59.2 (44.2–75.4)	60.7 (50.0–72.7)	75.0 (71.9–78.1)	52.2 (40.4–62.8)	59.2 (48.4–70.6)	67.1 (55.0–77.7)	60.2 (46.8–73.6)	58.2 (50.5–67.6)	57.2 (48.7–69.1)	71.1 (55.2–80.9)
Age <35 years, sum (%)		1,041 (17.2)	34 (2.4)	3,183 (12.7)	45 (5.4)	0 (0.0)	34 (13.7)	28 (6.4)	44 (2.7)	1,081 (10.0)	11 (4.9)	9 (5.5)	697 (5.5)
Sex, sum (%) female		3,801 (62.7)	987 (68.8)	14,488 (57.9)	512 (61.8)	1 (50.0)	165 (66.5)	244 (55.6)	969 (60.1)	6,199 (57.3)	129 (57.6)	108 (66.3)	8,535 (67.5)
Race, sum (%)	Caucasian	4,159 (68.6)	1,081 (75.3)	17,707 (70.8)	619 (74.8)	2 (100.0)	172 (69.4)	308 (70.2)	1,242 (77.0)	7,722 (71.3)	168 (75.0)	128 (78.5)	8,938 (70.7)
	African American	471 (7.8)	110 (7.7)	2,206 (8.8)	69 (8.3)	0 (0.0)	21 (8.5)	43 (9.8)	122 (7.6)	1,047 (9.7)	27 (12.1)	12 (7.4)	1,439 (11.4)
	Hispanic	536 (8.8)	84 (5.9)	1,901 (7.6)	49 (5.9)	0 (0.0)	10 (4.0)	26 (5.9)	90 (5.6)	691 (6.4)	7 (3.1)	8 (4.9)	1,098 (8.7)
	Asian	172 (2.8)	16 (1.1)	381 (1.5)	13 (1.6)	0 (0.0)	1 (0.4)	8 (1.8)	21 (1.3)	145 (1.3)	1 (0.4)	3 (1.8)	182 (1.4)
	Unknown	727 (12.0)	144 (10.0)	2,824 (11.3)	78 (9.4)	0 (0.0)	44 (17.7)	54 (12.3)	137 (8.5)	1,221 (11.3)	21 (9.4)	12 (7.4)	993 (7.8)
Geographic division, sum (%)	New England (CT, ME, MA, NH, RI, VT)	320 (5.3)	80 (5.6)	820 (3.3)	48 (5.8)	0 (0.0)	1 (0.4)	19 (4.3)	85 (5.3)	533 (4.9)	8 (3.6)	2 (1.2)	568 (4.5)
	Middle Atlantic (NJ, NY, PA)	328 (5.4)	80 (5.6)	844 (3.4)	54 (6.5)	1 (50.0)	3 (1.2)	23 (5.2)	81 (5.0)	734 (6.8)	13 (5.8)	16 (9.8)	646 (5.1)
	East North Central (IN, IL, MI, OH, WI)	1,138 (18.8)	255 (17.8)	4,049 (16.2)	82 (9.9)	1 (50.0)	13 (5.2)	48 (10.9)	201 (12.5)	1,503 (13.9)	31 (13.8)	19 (11.7)	1,974 (15.6)
	West North Central (IA, KS, MN, MO, NE, ND, SD)	759 (12.5)	165 (11.5)	2,183 (8.7)	56 (6.8)	0 (0.0)	13 (5.2)	36 (8.2)	122 (7.6)	879 (8.1)	9 (4.0)	6 (3.7)	1,098 (8.7)
	South Atlantic (DE, DC, FL, GA, MD, NC, SC, VA, WV)	1,111 (18.3)	305 (21.3)	5,660 (22.6)	265 (32.0)	0 (0.0)	104 (41.9)	112 (25.5)	413 (25.6)	3,377 (31.2)	85 (37.9)	81 (49.7)	3,489 (27.6)
	East South Central (AL, KY, MS, TN)	183 (3.0)	88 (6.1)	1,336 (5.3)	22 (2.7)	0 (0.0)	36 (14.5)	46 (10.5)	97 (6.0)	540 (5.0)	22 (9.8)	6 (3.7)	613 (4.8)
	West South Central (AR, LA, OK, TX)	606 (10.0)	151 (10.5)	3,713 (14.8)	51 (6.2)	0 (0.0)	44 (17.7)	44 (10.0)	146 (9.1)	507 (4.7)	20 (8.9)	10 (6.1)	1,783 (14.1)
	Mountain (AZ, CO, ID, NM, MT, UT, NV, WY)	595 (9.8)	141 (9.8)	2,684 (10.7)	128 (15.5)	0 (0.0)	26 (10.5)	49 (11.2)	245 (15.2)	1,563 (14.4)	28 (12.5)	12 (7.4)	1,341 (10.6)
	Pacific (AK, CA, HI, OR, WA)	999 (16.5)	167 (11.6)	3,569 (14.3)	120 (14.5)	0 (0.0)	8 (3.2)	58 (13.2)	214 (13.3)	1,137 (10.5)	6 (2.7)	11 (6.7)	1,086 (8.6)
	Unknown	26 (0.4)	3 (0.2)	161 (0.6)	2 (0.2)	0 (0.0)	0 (0.0)	4 (0.9)	8 (0.5)	53 (0.5)	2 (0.9)	0 (0.0)	52 (0.4)
*Time-varying covariates*													
Opioid average daily dose, median (Q1-Q3), MME		9.0 (4.5–18.0)	60.0 (60.0–120)	22.5 (15.0–32.1)	64.0 (48.0–96.0)	65.2 (44.0–65.2)	20.0 (10.0–30.0)	240 (80.0–600)	60.0 (43.5–120)	60.0 (30.0–90.0)	120 (60.0–180)	80.0 (60.0–120)	15.0 (10.0–20.0)
Unintentional traumatic injury, ever prior to the day of observation,^a^ person-days (%)		93,889 (71.7)	75,649 (79.3)	774,045 (72.6)	26,384 (81.8)	53 (100.0)	3,759 (49.1)	41,364 (78.5)	119,191 (78.3)	485,679 (75.8)	23,681 (82.7)	8,957 (88.9)	425,268 (74.1)
Unintentional traumatic injury, ever prior to the first day of observation,^a^ episodes (%)		3,491 (57.3)	902 (62.9)	13,137 (52.2)	550 (66.3)	2 (100.0)	132 (53.2)	277 (62.7)	972 (60.2)	6,074 (55.9)	161 (71.9)	122 (74.8)	7,494 (58.9)

a Diagnosis (any position, any claim type) ever prior to the day of observation.

Hydrocod, hydrocodone; hydromor, hydromorphone; levorph, levorphanol; MME, morphine milligram equivalents; oxymorph, oxymorphone; Q, quartile.

Before application of inclusion criteria, we identified 72,343, 51,012, and 54,089 sets of object + precipitant base pairs with candidate precipitants when the opioid objects were hydrocodone, tramadol, and oxycodone, respectively. After application of inclusion criteria, we examined 16,024 (22%), 8185 (16%), and 9330 (17%) sets of base pairs with candidate precipitants in confounder-adjusted self-controlled case series studies of unintentional traumatic injury. Table 2 provides summary data on RRs for unintentional traumatic injury, before and after confounder adjustment; Supplementary Tables S4, S5 provide summary data for typical hip fracture and motor vehicle crash, respectively. The volcano plots in Supplementary Figures S1, S2 graphically depict semi-Bayes shrunk confounder-adjusted RRs for the unintentional traumatic injury and typical hip fracture, respectively; corresponding secondary analyses using an alternate variance parameter for semi-Bayes shrinkage yielded similar findings (Supplementary Figures S3, S4, respectively). There were not any valid adjusted models generated for motor vehicle crash; thus, plots were not produced for this outcome.

**TABLE 2:** Summary data on rate ratios for unintentional traumatic injury, by object drug.

	Object Drug											
	Codeine	Fentanyl	Hydrocod	Hydromor	Levorph	Meperidine	Methadone	Morphine	Oxycodone	Oxymorph	Tapentadol	Tramadol
*Unadjusted* analyses, *before* semi-Bayes shrinkage												
Drug triplets examined, sum	4,029	2,303	16,751	695	NA	11	250	2,096	10,035	75	15	10,546
3DIs, sum (%)	231 (5.7)	250 (10.9)	1,089 (6.5)	20 (2.9)	NA	0 (0.0)	18 (7.2)	146 (7.0)	666 (6.6)	2 (2.7)	1 (6.7)	673 (6.4)
Increased rate^a^	87 (2.2)	38 (1.7)	409 (2.4)	11 (1.6)	NA	0 (0.0)	1 (0.4)	59 (2.8)	267 (2.7)	0 (0.0)	1 (6.7)	290 (2.7)
Decreased rate^b^	144 (3.6)	212 (9.2)	680 (4.1)	9 (1.3)	NA	0 (0.0)	17 (6.8)	87 (4.2)	399 (4.0)	2 (2.7)	0 (0.0)	383 (3.6)
RR geometric mean ± SD	0.84 ± 2.84	0.76 ± 2.92	0.87 ± 2.61	0.80 ± 3.03	NA	0.54 ± 2.51	0.61 ± 3.23	0.85 ± 3.00	0.85 ± 2.73	0.57 ± 2.75	2.00 ± 2.68	0.89 ± 2.64
RR range, min to max	0.00–85.20	0.02–51.33	0.02–78.22	0.05–25.05	NA	0.19–3.00	0.03–13.23	0.02–108.64	0.01–89.83	0.05–4.42	0.41–16.40	0.02–65.63
*Confounder-adjusted* analyses, *before* semi-Bayes shrinkage												
Drug triplets examined, sum	1,941	1,690	16,024	289	NA	NA	17	1,281	9,330	7	NA	8,185
3DIs, sum (%)	154 (7.9)	199 (11.8)	1,264 (7.9)	10 (3.5)	NA	NA	1 (5.9)	109 (8.5)	749 (8.0)	0 (0.0)	NA	659 (8.1)
Increased rate^a^	61 (3.1)	39 (2.3)	512 (3.2)	4 (1.4)	NA	NA	0 (0.0)	48 (3.7)	302 (3.2)	0 (0.0)	NA	296 (3.6)
Decreased rate^b^	93 (4.8)	160 (9.5)	752 (4.7)	6 (2.1)	NA	NA	1 (5.9)	61 (4.8)	447 (4.8)	0 (0.0)	NA	363 (4.4)
RR geometric mean ± SD	0.88 ± 3.00	0.78 ± 3.10	0.88 ± 2.77	0.81 ± 3.25	NA	NA	0.46 ± 2.93	0.88 ± 3.27	0.87 ± 2.88	0.80 ± 2.57	NA	0.92 ± 2.75
RR range, min to max	0.01–32.93	0.01–329.48	0.01–98.98	0.03–29.77	NA	NA	0.07–3.82	0.00–110.29	0.01–226.25	0.28–3.05	NA	0.01–185.41
*Unadjusted* analyses, *after* semi-Bayes shrinkage												
Drug triplets examined, sum	4,029	2,303	16,751	695	NA	11	250	2,096	10,035	75	15	10,546
3DIs, sum (%)	20 (0.5)	150 (6.5)	246 (1.5)	0 (0.0)	NA	0 (0.0)	9 (3.6)	19 (0.9)	128 (1.3)	0 (0.0)	0 (0.0)	126 (1.2)
Increased rate^a^	5 (0.1)	0 (0.0)	61 (0.4)	0 (0.0)	NA	0 (0.0)	0 (0.0)	4 (0.2)	32 (0.3)	0 (0.0)	0 (0.0)	42 (0.4)
Decreased rate^b^	15 (0.4)	150 (6.5)	185 (1.1)	0 (0.0)	NA	0 (0.0)	9 (3.6)	15 (0.7)	96 (1.0)	0 (0.0)	0 (0.0)	84 (0.8)
RR geometric mean ± SD	0.87 ± 1.23	0.68 ± 1.26	0.89 ± 1.26	0.85 ± 1.18	NA	0.46 ± 1.16	0.61 ± 1.22	0.87 ± 1.24	0.88 ± 1.26	0.53 ± 1.16	1.39 ± 1.21	0.93 ± 1.26
RR range, min to max	0.40–2.61	0.28–1.63	0.28–2.87	0.49–1.71	NA	0.37–0.62	0.29–0.97	0.40–2.49	0.20–2.76	0.35–0.73	0.86–1.87	0.30–3.10
*Confounder-adjusted* analyses, *after* semi-Bayes shrinkage												
Drug triplets examined, sum	1,941	1,690	16,024	289	NA	NA	17	1,281	9,330	7	NA	8,185
3DIs, sum (%)	13 (0.7)	108 (6.4)	241 (1.5)	0 (0.0)	NA	NA	0 (0.0)	11 (0.9)	145 (1.6)	0 (0.0)	NA	138 (1.7)
Increased rate^a^	4 (0.2)	0 (0.0)	75 (0.5)	0 (0.0)	NA	NA	0 (0.0)	5 (0.4)	42 (0.5)	0 (0.0)	NA	57 (0.7)
Decreased rate^b^	9 (0.5)	108 (6.4)	166 (1.0)	0 (0.0)	NA	NA	0 (0.0)	6 (0.5)	103 (1.1)	0 (0.0)	NA	81 (1.0)
RR geometric mean ± SD	0.90 ± 1.26	0.70 ± 1.28	0.91 ± 1.27	0.90 ± 1.20	NA	NA	0.47 ± 1.19	0.91 ± 1.26	0.89 ± 1.27	0.73 ± 1.11	NA	0.94 ± 1.28
RR range, min to max	0.35–3.05	0.28–1.74	0.29–2.75	0.60–2.06	NA	NA	0.32–0.67	0.42–2.64	0.20–2.86	0.62–0.87	NA	0.30–2.75

a Lower bound of the 95% confidence interval for the RR of interest excluded the null value.

b Upper bound of the 95% confidence interval for the RR of interest excluded the null value.

Hydrocod, hydrocodone; hydromor, hydromorphone; levorph, levorphanol; max, maximum; min, minimum; NA, not applicable; oxymorph, oxymorphone; RR, rate ratio; SD, standard deviation; 3DI, drug-drug-drug interaction.

**FIGURE 2:**
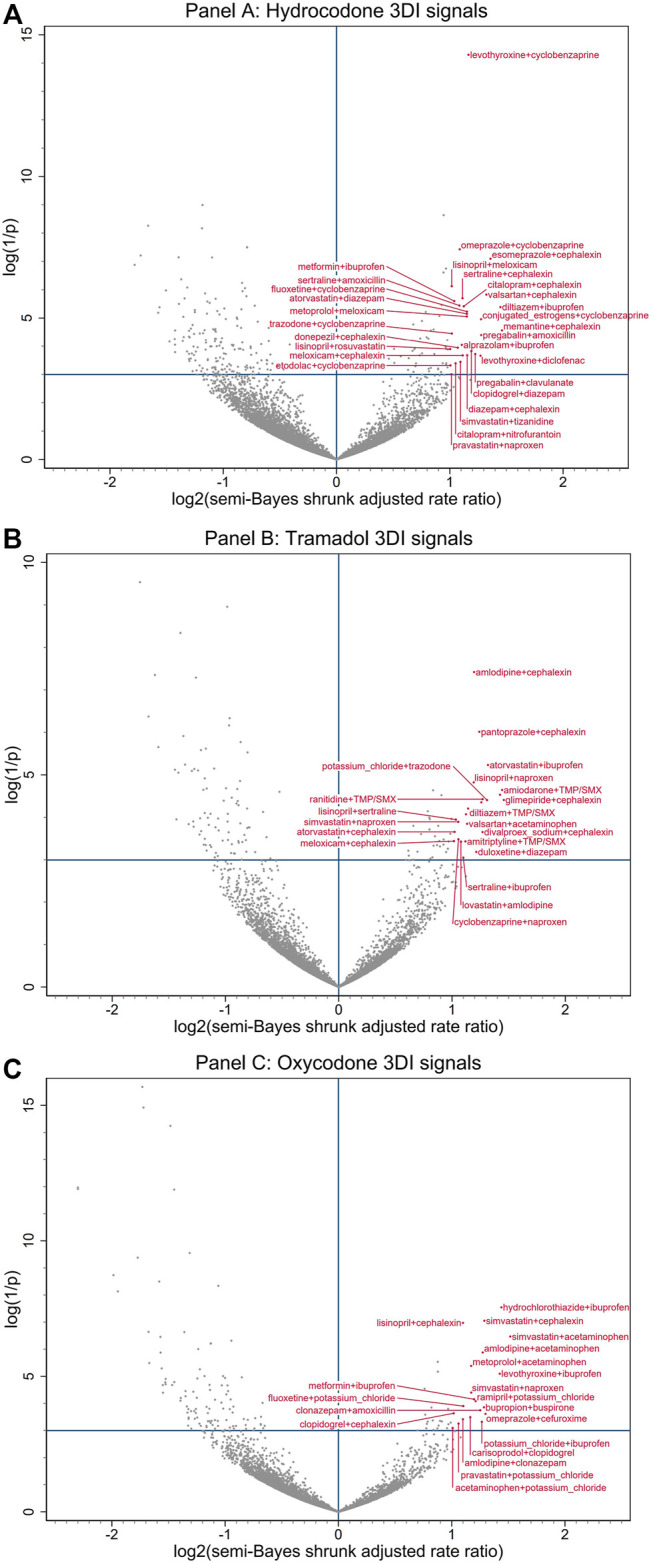
Commonly prescribed opioid + precipitant base pair with candidate interacting precipitant associations with unintentional traumatic injury. **(A)** depicts associations with hydrocodone. **(B)** depicts associations with tramadol. **(C)** depicts associations with oxycodone. Semi-Bayes shrinkage prespecified a variance of 0.25, assuming that 95% of true rate ratios would fall within an unspecified 7-fold range of each other. The *x*-axis represents the log base 2 semi-Bayes shrunk adjusted rate ratio for opioid + precipitant base pair with candidate interacting precipitant vs opioid + precipitant base pair. The *y*-axis represents the log(1/*p*-value) for the semi-Bayes shrunk adjusted rate ratio. Data points in the upper right quadrant represent drug triplets with a statistically significant signal for elevated risk of unintentional traumatic injury. For ease of reading, we limited labeling to upper right quadrant data points with log base 2 semi-Bayes shrunk adjusted rate ratio ≥1 or log(1/*p*-value) ≥10. We excluded signals with propoxyphene (a medical product eventually withdrawn from the United States market) from the plots, as they may have represented opioid switching rather than concomitant therapy. 3DI = drug-drug-drug interactions, SMX = sulfamethoxazole, TMP = trimethoprim.

Of 38,764 drug triplets across all assessed opioid objects, 183 (4.7 per thousand triplets) had statistically significantly elevated adjusted RRs for unintentional traumatic injury after semi-Bayes shrinkage. The majority of these were sets of base pairs with candidate precipitants for hydrocodone, tramadol, and oxycodone, which had 75 (0.5%), 57 (0.7%), and 42 (0.5%) statistically significantly elevated adjusted RRs for unintentional traumatic injury. We therefore deemed these 174 sets of object + precipitant base pairs with candidate precipitants (3 unique opioid objects, 50 unique base precipitants, 34 unique candidate precipitants) as potential 3DI signals (Table 3; Figure 2). The corresponding secondary analysis removing episodes with unintentional traumatic injury prior to the first day of observation yielded generally similar findings, with 34/7,918 (0.4%), 14/3,078 (0.5%), and 11/3,814 (0.3%) statistically significantly elevated adjusted RRs for hydrocodone, tramadol, and oxycodone (Supplementary Table S6).

**TABLE 3:** Drug-drug-drug interaction signals with statistically significantly increased rates of unintentional traumatic injury for commonly used opioids, by therapeutic category of base pair precipitant drug.

Object	Base Precipitant, Therapeutic Category	Base Precipitant, Drug	Candidate Interacting Precipitant, Drug	Rate Ratio, Semi-bayes Shrunk and Adjusted	95% Confidence Interval
HYDROCODONE	Anti-infective	amoxicillin	ibuprofen	1.92	1.05–3.49
	Cardiovascular	amlodipine	cephalexin	1.95	1.30–2.93
		amlodipine	sulfamethoxazole	1.81	1.15–2.85
		amlodipine	trimethoprim	1.76	1.12–2.75
		atenolol	cyclobenzaprine	1.88	1.19–2.95
		atorvastatin	cephalexin	1.55	1.02–2.36
		atorvastatin	diazepam	2.22	1.26–3.92
		diltiazem	cephalexin	1.76	1.02–3.01
		diltiazem	cyclobenzaprine	1.95	1.12–3.41
		**diltiazem**	**ibuprofen**	**2.73** **^a^** ^**,**^ **^b^**	**1.36–5.45**
		lisinopril	cephalexin	1.42	1.03–1.97
		lisinopril	meloxicam	2.02	1.29–3.18
		lisinopril	rosuvastatin	2.00	1.11–3.61
		lisinopril	sulfamethoxazole	1.59	1.07–2.37
		lisinopril	trimethoprim	1.56	1.05–2.31
		lovastatin	cyclobenzaprine	1.95	1.11–3.44
		metoprolol	diazepam	1.76	1.01–3.06
		metoprolol	meloxicam	2.22	1.25–3.94
		metoprolol	prednisone	1.60	1.13–2.27
		metoprolol	sulfamethoxazole	1.73	1.17–2.54
		metoprolol	trimethoprim	1.69	1.15–2.47
		pravastatin	cyclobenzaprine	1.86	1.03–3.38
		pravastatin	naproxen	2.02	1.00–4.08
		simvastatin	naproxen	1.74	1.12–2.69
		simvastatin	tizanidine	2.13	1.07–4.27
		**valsartan**	**cephalexin**	**2.50**	**1.37–4.58**
		valsartan	sulfamethoxazole	1.90	1.01–3.55
		valsartan	trimethoprim	1.90	1.01–3.55
	Central nervous system	alprazolam	ibuprofen	2.15	1.14–4.05
		citalopram	cephalexin	2.18	1.27–3.72
		citalopram	cyclobenzaprine	1.66	1.07–2.60
		citalopram	nitrofurantoin	2.07	1.06–4.06
		cyclobenzaprine	cephalexin	1.78	1.03–3.09
		cyclobenzaprine	diazepam	1.78	1.14–2.77
		cyclobenzaprine	prednisone	1.42	1.06–1.91
		diazepam	cephalexin	2.22	1.10–4.48
		donepezil	cephalexin	2.10	1.13–3.91
		etodolac	cyclobenzaprine	2.01	1.05–3.86
		fluoxetine	cyclobenzaprine	2.22	1.27–3.89
		fluoxetine	ibuprofen	1.95	1.05–3.64
		gabapentin	cephalexin	1.63	1.04–2.55
		meloxicam	cephalexin	2.16	1.10–4.26
		**memantine**	**cephalexin**	**2.75**	**1.27–5.97**
		**pregabalin**	**amoxicillin**	**2.42** **^a^** ^**,**^ **^b^**	**1.21–4.85**
		**pregabalin**	**clavulanate**	**2.34** **^a^** ^**,**^ **^b^**	**1.12–4.89**
		sertraline	amoxicillin	2.12	1.27–3.55
		sertraline	cephalexin	2.16	1.29–3.62
		trazodone	cyclobenzaprine	2.03	1.17–3.50
		zolpidem	diazepam	1.98	1.11–3.52
	Endocrine and metabolic	**conjugated estrogens**	**cyclobenzaprine**	**2.42** **^a^** ^**,**^ **^b^**	**1.27–4.61**
		levothyroxine	cephalexin	1.48	1.05–2.10
		levothyroxine	cyclobenzaprine	2.24	1.63–3.08
		**levothyroxine**	**diclofenac**	**2.41**	**1.11–5.24**
		levothyroxine	isosorbide mononitrate	1.96	1.05–3.64
		levothyroxine	meloxicam	1.82	1.08–3.06
		metformin	cephalexin	1.92	1.29–2.87
		metformin	ibuprofen	2.06	1.26–3.34
		metformin	sulfamethoxazole	1.60	1.01–2.54
		metformin	trimethoprim	1.60	1.01–2.54
	Gastrointestinal	**esomeprazole**	**cephalexin**	**2.57**	**1.48–4.46**
		omeprazole	cephalexin	1.67	1.11–2.51
		omeprazole	cyclobenzaprine	2.12	1.38–3.27
		omeprazole	naproxen	1.95	1.12–3.39
		omeprazole	sulfamethoxazole	1.69	1.07–2.66
		omeprazole	trimethoprim	1.62	1.03–2.55
		pantoprazole	duloxetine	1.97	1.04–3.75
	Hematological	**clopidogrel**	**diazepam**	**2.28** **^a^** ^**,**^ **^c^**	**1.13–4.62**
	Nutrients and nutritional	potassium chloride	cyclobenzaprine	1.90	1.22–2.96
	Renal and genitourinary	furosemide	clindamycin	1.86	1.02–3.39
		furosemide	sulfamethoxazole	1.62	1.08–2.42
		furosemide	trimethoprim	1.61	1.08–2.41
		hydrochlorothiazide	cephalexin	1.93	1.37–2.71
		hydrochlorothiazide	cyclobenzaprine	1.38	1.03–1.83
	Respiratory	montelukast	cyclobenzaprine	1.97	1.14–3.40
OXYCODONE	Cardiovascular	**amlodipine**	**Acetaminophen**	**2.42**	**1.36–4.31**
		amlodipine	cephalexin	1.71	1.01–2.90
		amlodipine	ciprofloxacin	1.78	1.04–3.04
		amlodipine	clonazepam	2.14	1.06–4.31
		amlodipine	potassium chloride	1.72	1.03–2.87
		lisinopril	cephalexin	2.14	1.36–3.37
		**metoprolol**	**Acetaminophen**	**2.25**	**1.28–3.95**
		metoprolol	cephalexin	1.73	1.04–2.87
		metoprolol	potassium chloride	1.84	1.19–2.83
		pravastatin	potassium chloride	2.09	1.04–4.18
		**ramipril**	**potassium chloride**	**2.31** **^a^** ^**,**^ **^b^**	**1.16–4.61**
		**simvastatin**	**Acetaminophen**	**2.86**	**1.49–5.49**
		**simvastatin**	**cephalexin**	**2.44**	**1.44–4.13**
		simvastatin	ibuprofen	1.99	1.04–3.80
		**simvastatin**	**naproxen**	**2.25**	**1.19–4.26**
		simvastatin	potassium chloride	1.70	1.06–2.73
	Central nervous system	Acetaminophen	potassium chloride	2.01	1.01–3.99
		**bupropion**	**buspirone**	**2.44** **^c^**	**1.14–5.19**
		bupropion	cyclobenzaprine	1.96	1.03–3.72
		carisoprodol	clopidogrel	2.24	1.08–4.65
		**clonazepam**	**amoxicillin**	**2.38** **^a^** ^**,**^ **^b^**	**1.12–5.05**
		diazepam	prednisone	1.79	1.00–3.19
		fluoxetine	potassium chloride	2.15	1.13–4.09
		ibuprofen	methylprednisolone	1.83	1.01–3.32
	Endocrine and metabolic	levothyroxine	ciprofloxacin	1.84	1.21–2.78
		levothyroxine	clonazepam	1.94	1.01–3.73
		levothyroxine	cyclobenzaprine	1.77	1.09–2.87
		**levothyroxine**	**ibuprofen**	**2.68**	**1.32–5.43**
		metformin	cephalexin	1.96	1.10–3.48
		**metformin**	**ibuprofen**	**2.29** **^a^**	**1.17–4.50**
	Gastrointestinal	**omeprazole**	**cefuroxime**	**2.46** **^a^**	**1.11–5.46**
		omeprazole	hydrochlorothiazide	1.86	1.04–3.32
		omeprazole	ibuprofen	1.88	1.04–3.40
		omeprazole	potassium chloride	1.62	1.00–2.63
	Hematological	clopidogrel	cephalexin	2.02	1.09–3.77
		warfarin	cephalexin	1.85	1.02–3.33
	Nutrients and nutritional	**potassium chloride**	**ibuprofen**	**2.41**	**1.06–5.47**
	Renal and genitourinary	hydrochlorothiazide	cyclobenzaprine	1.69	1.13–2.54
		**hydrochlorothiazide**	**ibuprofen**	**2.71** **^a^** ^**,**^ **^c^**	**1.54–4.76**
TRAMADOL	Cardiovascular	**amiodarone**	**sulfamethoxazole**	**2.68** **^c^**	**1.26–5.73**
		**amiodarone**	**trimethoprim**	**2.72** **^c^**	**1.27–5.79**
		**amlodipine**	**cephalexin**	**2.29**	**1.43–3.67**
		amlodipine	ibuprofen	1.98	1.08–3.62
		amlodipine	nitrofurantoin	1.90	1.06–3.40
		amlodipine	ondansetron	1.80	1.06–3.05
		atorvastatin	cephalexin	2.03	1.09–3.79
		atorvastatin	clavulanate	1.96	1.06–3.62
		**atorvastatin**	**ibuprofen**	**2.49**	**1.31–4.74**
		diltiazem	hydrochlorothiazide	1.90	1.05–3.44
		diltiazem	sulfamethoxazole	2.18	1.15–4.13
		diltiazem	trimethoprim	2.21	1.17–4.18
		**lisinopril**	**naproxen**	**2.28** **^a^** ^**,**^ **^b^**	**1.24–4.21**
		lisinopril	sertraline	2.05	1.12–3.73
		lisinopril	sulfamethoxazole	1.75	1.10–2.79
		lisinopril	trimethoprim	1.74	1.10–2.76
		lovastatin	amlodipine	2.11	1.06–4.19
		metoprolol	Acetaminophen	1.84	1.09–3.10
		metoprolol	amoxicillin	1.87	1.16–3.04
		metoprolol	cephalexin	1.54	1.01–2.34
		metoprolol	clavulanate	1.75	1.00–3.06
		metoprolol	sulfamethoxazole	1.62	1.02–2.57
		simvastatin	naproxen	2.08	1.12–3.85
		simvastatin	nitrofurantoin	1.86	1.04–3.32
		valsartan	Acetaminophen	2.19	1.12–4.28
	Central nervous system	amitriptyline	sulfamethoxazole	2.17	1.07–4.41
		amitriptyline	trimethoprim	2.17	1.07–4.41
		cyclobenzaprine	naproxen	2.08	1.07–4.04
		**divalproex sodium**	**cephalexin**	**2.41**	1.11–5.23
		**duloxetine**	**diazepam**	**2.32** **^c^** ^**,**^ **^d^**	1.03–5.21
		gabapentin	alprazolam	1.85	1.05–3.25
		meloxicam	cephalexin	2.02	1.06–3.85
		sertraline	ibuprofen	2.14	1.01–4.55
		sertraline	sulfamethoxazole	1.95	1.04–3.67
		sertraline	trimethoprim	1.95	1.04–3.67
	Endocrine and metabolic	**glimepiride**	**cephalexin**	**2.75**	1.25–6.05
		levothyroxine	cephalexin	1.51	1.00–2.27
		levothyroxine	sulfamethoxazole	1.58	1.00–2.48
		levothyroxine	trimethoprim	1.66	1.06–2.59
		metformin	sulfamethoxazole	1.77	1.01–3.12
		metformin	trimethoprim	1.83	1.04–3.20
	Gastrointestinal	omeprazole	furosemide	1.58	1.07–2.34
		**pantoprazole**	**cephalexin**	**2.36**	1.35–4.12
		**ranitidine**	**sulfamethoxazole**	**2.39** **^b^**	1.20–4.76
		**ranitidine**	**trimethoprim**	**2.41** **^b^**	1.21–4.79
	Hematological	clopidogrel	cephalexin	1.93	1.03–3.63
	Nutrients and nutritional	**potassium chloride**	**trazodone**	**2.48** **^a^** ^**,**^ **^b^**	1.22–5.04
		potassium chloride	trimethoprim	1.79	1.04–3.07
	Renal and genitourinary	furosemide	cephalexin	1.53	1.02–2.30
		furosemide	sulfamethoxazole	1.72	1.11–2.68
		furosemide	trimethoprim	1.78	1.15–2.76
		hydrochlorothiazide	naproxen	1.99	1.12–3.55
		hydrochlorothiazide	sulfamethoxazole	1.74	1.07–2.83
		hydrochlorothiazide	trimethoprim	1.74	1.07–2.83

a Drug interactions involving only two of the drugs in the drug triplet documented in Facts & Comparisons Clinical Drug Information (Wolters Kluwer: Alphen aan den Rijn, South Holland, Netherlands).

b Drug interactions involving all three of the drugs in the drug triplet documented in Facts & Comparisons Clinical Drug Information (Wolters Kluwer: Alphen aan den Rijn, South Holland, Netherlands).

c Drug interactions involving only two of the drugs in the drug triplet documented in Micromedex (IBM, Watson Health: Cambridge, Massachusetts, United States).

d Drug interactions involving all three of the drugs in the drug triplet documented in Micromedex (IBM, Watson Health: Cambridge, Massachusetts, United States).

**Bolded** rows indicate 3DI signals with rate ratio ≥2.25.

The most common putative interacting combinations of base precipitants with candidate precipitants were cardiovascular with anti-infective agents (*N* = 33, including 10 calcium channel blockers with antibiotics), cardiovascular with CNS agents (*N* = 31, including 7 statins with nonopioid analgesics), CNS with anti-infective agents (*N* = 19, including 8 antidepressants with antibiotics), CNS with CNS agents (*N* = 14, including 4 antidepressants with skeletal muscle relaxants), endocrine/metabolic with anti-infective agents (*N* = 12, including 7 antidiabetics with antibiotics), and endocrine/metabolic with CNS agents (*N* = 10, including 3 thyroid hormones with nonopioid analgesics). The highest magnitude statistically significantly elevated adjusted RRs for unintentional traumatic injury after semi-Bayes shrinkage were 2.86 (95% CI: 1.49–5.49) for oxycodone + simvastatin with acetaminophen, 2.75 (1.27–5.97) for hydrocodone + memantine with cephalexin, and 2.75 (1.25–6.05) for tramadol + glimepiride with cephalexin. In terms of interaction reporting in Micromedex and/or Facts & Comparisons for the 34 highest magnitude 3DI signals (RR ≥ 2.25), 12 (35.3%) had reporting for drug interactions involving two of the drugs in the drug triplets and 6 (17.6%) had reporting for drug interactions involving all three of the drugs in the drug triplets (Table 3).

## 4 Discussion

Higher-order pharmacokinetic and pharmacodynamic drug interactions have long been recognized, yet remain poorly understood from a clinical perspective due to a lack of study (Horn and Hansten, 2011). In this paper, we apply a novel approach for semi-automated, high throughput screening of administrative healthcare data to identify potential real-world 3DIs, generating hypotheses that can provide a foundation for crucially needed 3DI etiologic studies. As the physiologic/metabolic characteristics of opioids and the common co-prescription profiles among opioid users yield a particularly high propensity for 3DIs, we built on our prior screening study of pairwise opioid DDIs to assess for 3DI signals associated with an increased rate of unintentional traumatic injury (Leonard et al., 2020). Among 38,764 drug triplets including an opioid object, we identified 183 (4.7 per thousand triplets) statistically significantly elevated adjusted RRs for unintentional traumatic injury. Of these, 174 (95%) triplets involved the three most commonly dispensed opioids (hydrocodone, tramadol, and oxycodone). Although associations with hip fracture and motor vehicle crash were also assessed, no 3DI signals were identified for these prespecified subsets of unintentional traumatic injury.

The limited pool of prior epidemiologic evidence, as well as the predominance of mechanism-based ideas about opioid 3DIs, have centered on enhanced CNS depression from concurrent use of three CNS-active agents, with particular focus on the “holy trinity” of opioids with benzodiazepines and skeletal muscle relaxants (Forrester, 2011; Garg et al., 2017; Horsfall and Sprague, 2017; Wang et al., 2019; Li et al., 2020; Watanabe and Yang, 2020b, 2020a). In our study, the mechanism of injury that would be anticipated to underlie many of the observed interaction signals would be these additive or synergistic pharmacodynamic effects from concomitant use of three CNS depressants. Specifically, we found concomitant use to be associated with injury rates potentially increased by: 1.8-fold for hydrocodone + cyclobenzaprine (*skeletal muscle relaxant*) with diazepam (*benzodiazepine*) (vs hydrocodone + cyclobenzaprine alone); 1.9-fold for tramadol + gabapentin (*anticonvulsant*) with alprazolam (*benzodiazepine*); and 2.0-fold for hydrocodone + zolpidem (*hypnotic*) with diazepam (*benzodiazepine*). These findings are consistent with recent studies that have found an increased risk of opioid overdose emergency department visits/hospitalizations (Li et al., 2020), all-cause emergency department visits (Watanabe and Yang, 2020a), all-cause hospitalizations (Watanabe and Yang, 2020b), and opioid overdose deaths (Garg et al., 2017) associated with the concurrent use of opioid, benzodiazepines, and skeletal muscle relaxants. The biological plausibility of the observed interaction signals, together with prior epidemiologic evidence, supports the validity and utility of our screening approach for detecting clinically meaningful 3DIs. The absence of statistically significant signals for related combinations of opioids with CNS depressants (e.g., the previously highlighted opioid-benzodiazepine interactions with carisoprodol (Horsfall and Sprague, 2017; Li et al., 2020)) may reflect the limited statistical precision inherent in the lower numbers of concurrent dispensations for three potentially interacting drugs, and/or our selection of conservative assumptions when adjusting for multiple estimation using the semi-Bayes method. The latter point was essential to our semi-automated screening approach, as the intent was to generate hypotheses for future etiologic studies. Therefore, we deliberately set parameters that we realized would reduce sensitivity (i.e., permitting some false negatives) because we placed greater value on achieving higher specificity (i.e., minimizing false positives).

The majority of potential 3DIs flagged by our screening have not been described in the literature, although many have plausible physiologic, biochemical, and/or metabolic pathways. A number of these detected 3DIs involve mechanisms that warrant further investigation given their high clinical importance and possible associations with unintentional traumatic injury risk. First, the potentiation of well-demonstrated pairwise interactions between opioids and CNS depressants via pharmacokinetic interactions that increase the serum concentration of the active opioid form, such as potent CYP2D6 inhibition by bupropion increasing the level of oxycodone (Kotlyar et al., 2005; Huddart et al., 2018), and potentially enhancing its pharmacodynamic interactions (e.g., oxycodone + bupropion with cyclobenzaprine, RR_injury_ = 2.0; and oxycodone + bupropion with buspirone, RR_injury_ = 2.4). Second, the modulation of synaptic serotonin levels by select opioids, as well as many other CNS depressants (Scotton et al., 2019; Baldo and Rose, 2020), raises concerns that co-prescribing may elevate the risk of potentially injury-inducing CNS depression and serotonin toxicity (e.g., hydrocodone + citalopram with cyclobenzaprine, RR_injury_ = 1.7; hydrocodone + trazodone with cyclobenzaprine, RR_injury_ = 2.0; and hydrocodone + fluoxetine with cyclobenzaprine, RR_injury_ = 2.2; however, the specific serotonergic effects of hydrocodone and cyclobenzaprine remain controversial and merit further assessment (Gillman, 2009; Baldo and Rose, 2020)). While the central symptoms of serotonin toxicity (i.e., altered mental status, autonomic nervous system overactivity, and neuromuscular hyperactivity) may contribute to traumatic injury risk (Scotton et al., 2019; Baldo and Rose, 2020), the listed 3DI signal may also be reasonably attributed solely to the CNS depressant effects of the drug triplets. Third, hypotensive and orthostatic effects from drugs that are intended to lower blood pressure (e.g., beta blockers, calcium channel blockers) may compound CNS depression from opioid interactions with benzodiazepines or skeletal muscle relaxants (e.g., hydrocodone + metoprolol with diazepam, RR_injury_ = 1.8; hydrocodone + atenolol with cyclobenzaprine, RR_injury_ = 1.9; hydrocodone + diltiazem with cyclobenzaprine, RR_injury_ = 2.0; oxycodone + amlodipine with clonazepam, RR_injury_ = 2.1).

A substantial subset of the detected 3DI signals involved concurrent use of opioids with antibiotics and/or nonopioid analgesics. In select cases, prior research into pairwise interactions of these drugs has demonstrated plausible mechanisms for observed increases in injury risk. For example, in the case of the signal for oxycodone + amlodipine with ciprofloxacin (RR_injury_ = 1.8), CYP3A4 inhibition by ciprofloxacin may raise levels of both oxycodone and amlodipine (McLellan et al., 1996; Zhu et al., 2014; Huddart et al., 2018), potentially enhancing their pharmacodynamic interaction. However, in general, the relatively common signals among these drug classes are noteworthy as the mechanisms by which they would be interacting with opioids are unclear. While the absence of current mechanistic support does not preclude the possibility of unrecognized 3DI pathways, exploration of alternate explanations for these signals is warranted. Potential for within-person confounding by indication merits consideration, given the underlying factors in traumatic injury may also be related to prior infection and pain (for which antibiotics and opioid/nonopioid analgesics would respectively be prescribed). Thus, the observed associations may be partially attributable to the indications for specific drug combinations rather than their interactions (Maclure et al., 2012; Hennessy et al., 2016). Similarly, for initially less severe traumatic injuries (i.e., injuries for which an emergency department visit or hospitalization may not initially be sought), protopathic bias may play a role if the early symptoms of the injury included the pain that led to prescription of an opioid/nonopioid analgesic (Maclure et al., 2012).

An alternative explanation for 3DI signals with one of the involved drugs lacking a recognized interaction mechanism (i.e., many of the signals involving antibiotics and nonopioid analgesics), may relate to signal detection being defined by injury rates during time using opioid-precipitant base pairs when exposed vs. unexposed to a candidate interacting precipitant. This analytic approach introduces the possibility that some of the signals generated may represent potent pairwise DDIs between exclusively the opioid object and the candidate interacting precipitant, rather than true 3DIs (i.e., the base pair precipitant would have no true role in the interaction). However, as shown in Table 3, the 3DIs signals involving antibiotics and nonopioid analgesics predominantly have these drugs acting as the candidate interacting precipitant or as both precipitants, suggesting that this explanation would not be applicable to many of the observed 3DI signals without known interaction mechanisms.

Our study has notable strengths that make it a valuable foundation for future research on 3DIs. First, research on higher-order drug interactions has become increasingly vital in the context of highly prevalent and rising polypharmacy across the globe (Wastesson et al., 2018; World Health Organization, 2019). In this study, we developed and applied a novel approach to screen for 3DI signals in large-scale administrative healthcare data, without the need for *a priori* hypotheses regarding anticipated interactions. Second, we executed our 3DI screen utilizing a self-controlled case series design, which has been well-demonstrated as an effective screening strategy in the drug-drug (i.e., pairwise) interaction literature (Han et al., 2017). Importantly, this case-only approach eliminates between-person confounding and within-person confounding by time-invariant characteristics (Maclure et al., 2012; Hennessy et al., 2016). Moreover, our bi-directional implementation of this design reduced its susceptibility to exposure-trend bias (Maclure et al., 2012). Third, we focused this 3DI investigation on the known associations between opioid drug interactions and unintentional traumatic injury (Leonard et al., 2020), a topic of high clinical relevance with an outcome that has well-supported algorithms for classification in claims data. Finally, we maximized the specificity of our screening by using semi-Bayes shrinkage to account for multiple estimation.

Our study has limitations that are critical to recognize when interpreting the identified 3DI signals. First, claims for drug dispensations may not reflect drug intake. Second, the self-controlled case series design may be more susceptible to reverse causation than alternate self-controlled designs (though these alternate designs have their own limitations, including greater susceptibility to time trend bias) (Maclure et al., 2012). Third, it is possible that some of the signals generated may represent pairwise interactions between just one of the drugs in the base pair and the candidate interacting precipitant, rather than true 3DIs. Fourth, some of the medications studied as precipitants are available over-the-counter; thus, their concurrent use with opioids may have been poorly captured in claims data. Fifth, while we included opioid daily dose in our analysis as a time-varying covariate, there are multiple approaches that could be taken to further account for the effects of opioid doses over time on injury risk. Future etiological studies of detected opioid 3DI signals (intended to assess causation) may be strengthened by accounting for the effects of cumulative opioid doses, with approaches to flexible modeling of cumulative drug doses being increasingly well-validated (Sylvestre and Abrahamowicz, 2009; Danieli and Abrahamowicz, 2019; Li et al., 2022). Sixth, the assessed injury outcomes have not been specifically validated in the utilized data source (Optum), which limits the ability to interpret the completeness and accuracy of data capture. Finally, the detected 3DI signals may not generalize to populations beyond the assessed commercially insured and Medicare Advantage ambulatory care beneficiaries.

In summary, our study demonstrated the use of a pioneering semi-automated, high-throughput approach for detecting three-way drug interactions in large-scale administrative healthcare databases. We have applied this approach to identify signals of increased risk of unintentional traumatic injury related to 3DIs between prescription opioids and commonly co-prescribed medications. We identified 174 potential 3DI signals involving the three most commonly used opioids (75 for hydrocodone, 57 for tramadol, and 42 for oxycodone) based on their statistically significantly elevated adjusted RRs after semi-Bayes shrinkage. Review of prior pharmacokinetic and clinical evidence suggests that biologically plausible mechanisms of interaction underlie many of these observed 3DI signals, supporting the validity of our screening strategy. Building forward, the 3DI signals found in our screening provide important targets to guide future etiological investigations into higher-order opioid interactions and unintentional traumatic injury risk. Moreover, this novel 3DI screening method may be a valuable tool to advance research into higher-order interactions across different drug classes, clinical fields, and healthcare databases.

## Data Availability

The data analyzed in this study is subject to the following licenses/restrictions: The data that support the findings of this study are available from Optum Inc., but restrictions apply to the availability of these data, which were used under license for the current study and so are not publicly available. Data may be available from the authors upon reasonable request and with explicit permission from Optum Inc. Requests to access these datasets should be directed to Charles E. Leonard, celeonar@pennmedicine.upenn.edu.
