# Supplementary material for: Opioid Drug-Drug-Drug Interactions and Unintentional Traumatic Injury: Screening to Detect Three-Way Drug Interaction Signals
Source: Front Pharmacol. 2022 May 10;13:845485. doi: 10.3389/fphar.2022.845485 (PMC9127150; doi:10.3389/fphar.2022.845485)
Supplement: Supplementary file 1 [file DataSheet1.docx]

**SUPPLEMENTARY MATERIAL**

1. Supplementary Methods……………....…………………………………………… *Page 3*
2. Supplementary Tables
   1. Supplementary Table S1. Outcome definitions.…………..……………….. *Page 5*
   2. Supplementary Table S2. Descriptors of persons experiencing typical hip fracture while receiving opioid object drugs…………....……... *Page 9*
   3. Supplementary Table S3. Descriptors of persons experiencing motor vehicle crash (in which the subject was driving) while receiving opioid object drugs…….……………………………………..… *Page 12*
   4. Supplementary Table S4. Summary data on rate ratios for typical hip fracture, by object drug……………………………………..… *Page 15*
   5. Supplementary Table S5. Summary data on rate ratios for motor vehicle crash (in which the subject was driving), by object drug…………………………………………………………….. *Page 17*
   6. Supplementary Table S6. Drug-drug-drug interaction signals with statistically significantly increased rates of unintentional traumatic injury for commonly used opioids, by therapeutic category of base pair precipitant drug | Secondary analysis removing episodes with unintentional traumatic injury prior to the first day of observation………………………………………………………………... *Page 18*
3. Supplementary Figures
   1. Supplementary Figure S1. Volcano plot depicting opioid + precipitant base pair with candidate interacting precipitant associations with unintentional traumatic injury……………………….... *Page 20*
   2. Supplementary Figure S2. Volcano plot depicting opioid + precipitant base pair with candidate interacting precipitant associations with typical hip fracture.………………...………………….. *Page 26*
   3. Supplementary Figure S3. Volcano plot depicting opioid + precipitant base pair with candidate interacting precipitant associations with unintentional traumatic injury | Prespecified secondary analysis increasing semi-Bayes shrinkage variance parameter…...…………………………………………………………….. *Page 27*
   4. Supplementary Figure S4. Volcano plot depicting opioid + precipitant base pair with candidate interacting precipitant associations with typical hip fracture | Prespecified secondary analysis increasing semi-Bayes shrinkage variance parameter.………..… *Page 36*

**Supplementary Methods**

***Data source***

We utilized Optum’s de-identifed Clinformatics® Data Mart Database (Optum Inc, 2014) administrative data from May 1, 2000 through September 30, 2015. Optum includes longitudinal enrollment and healthcare billing data from >71 million commercially insured and Medicare Advantage beneficiaries of the largest US-based private health insurer by market share (Statista, 2019).

Optum’s de-identifed Clinformatics® Data Mart Database (Optum Inc, 2014) data elements include: demographics (e.g., age, sex, race); enrollment periods; medical encounters (e.g., ambulatory care visits, emergency department visits, inpatient hospitalizations) and their accompanying diagnoses and procedures; pharmacy dispensings in ambulatory care settings; and laboratory orders and results for a subset of beneficiaries. We selected Optum as our data source because of its generalizability to the United States population, as ~65% of Americans receive healthcare coverage via commercial health plans or Medicare.

Additional detail on Optum data is available at

<https://www.optum.com/content/dam/optum/resources/productSheets/Clinformatics_for_Data_Mart.pdf>.

***Identifying base pair precipitant drugs and candidate interacting precipitant drugs during opioid object drug use***

We used pharmacy claim dates and days’ supply values to identify prescription dispensings for the following opioids as object drugs of interest: codeine (including as an ingredient in cough medications); fentanyl; hydrocodone (including as an ingredient in cough medications); hydromorphone; levorphanol; meperidine; methadone; morphine; oxycodone; oxymorphone; tapentadol; and tramadol. We limited dispensings to the most commonly used route of administration per opioid (e.g., oral hydrocodone, transdermal fentanyl). During periods of apparent opioid use with an outcome, we used pharmacy claim dates and days’ supply values to identify prescription dispensings for any orally administered non-opioid concomitant drugs. These drugs functioned as the list of potential base pair precipitants, which was then limited to those drugs with at least 5 exposed patients during their opioid observation time. Among those patients with an outcome during their first concomitant opioid + precipitant base pair exposure time, candidate interacting precipitants were similarly specified based on the list of prescription dispensings for orally administered non-opioid concomitant drugs. Using this process, concomitant drugs could potentially function as a base pair precipitant or candidate interacting precipitant in different drug triplets; however, the observation time and the causal contrast would differ depending on whether specified as the base pair precipitant or candidate interacting precipitant. We linked national drug codes to the Lexicon Plus Drug Database (Cerner Multum: Denver, CO, US) to categorize candidate precipitants by medication class.

***Bi-directionality in the context of a self-controlled case series study***

In our application of the self-controlled case series design, the focal window is comprised of person-days exposed to the object + precipitant base pairs and the candidate interacting precipitant. The referent windows are comprised of person-days exposed to the object + precipitant base pairs only (i.e., person-days unexposed to the candidate interacting precipitant). Our permitting a referent window before and after the focal window is indicative of a bi-directional implementation of the self-controlled case series design. This is the design’s standard implementation.

***Morphine milligram equivalent (MME) calculation***

We calculated MME as (((strength per unit / unit) x quantity) / days’ supply) x (conversion factor for opioid of interest). We used the MME conversion list (v.02/2018) published by the Centers for Medicare and Medicaid Services. For example, a prescription claim for the combination product hydrocodone/acetaminophen 10mg/325 mg tablets, quantity dispensed = 100, and days’ supply = 25 would have MME dose = (((10 / 1) x 100) / 25) x 1 = 40. A prescription claim for fentanyl 25 microgram/hour patches, quantity dispensed = 10, and DS = 30 would have MME dose = (((25 / 1) x 10) / 30) x 7.2 = 60. In consultation with pain medicine physicians of our health system, we set the maximum permissible MME dose value to 1,500. If MME dose was calculated as >1,500, we then set it to 1,500. We included MME in regression models as follows: quartile; if not, binary; if not, covariate dropped.

***Semi-Bayes shrinkage***

Operationally, we prespecified a variance (σ2 = 0.25) to assume that 95% of true rate ratios would be within an unspecified 7-fold range of each other (e.g., 0.5–3.5, since stronger drug interaction associations would likely have already been identified via case reports), then shrunk outlying effect estimates toward their geometric mean. In a prespecified secondary analysis, we increased the variance (σ2 = 0.67) to assume that 95% of true rate ratios would be within an unspecified 25-fold range of each other.

***References***

Optum Inc (2014). Clinformatics Data Mart. Available at: https://www.optum.com/content/dam/optum/resources/productSheets/Clinformatics_for_Data_Mart.pdf [Accessed June 13, 2020].

Statista (2019). Market share of leading health insurance companies in the United States in 2018, by direct premiums written. Available at: https://www.statista.com/statistics/216518/leading-us-health-insurance-groups-in-theus/ [Accessed June 13, 2020].

**Supplementary Table S1. Outcome definitions**

| **Priority** | **Outcome** | **Discharge diagnosis descriptor** | **Discharge diagnosis ICD-9-CM code(s)** | **Discharge diagnosis position and claim type** | **Performance characteristics and/or other supporting information** |
| --- | --- | --- | --- | --- | --- |
| Primary | Unintentional traumatic injury | fracture | 800–829 | Any-position discharge diagnosis on an ED claim  or  Principal inpatient discharge diagnosis on an inpatient hospitalization claim | Adapted from the injury definition used by the American College of Surgeons’ NTDB Data Standard (version 03.2015).  NTDB’s injury definition explicitly excludes the following: late effects of injuries, poisonings, toxic effects, and other external causes; superficial injury; contusion with intact skin surface; and effects of a foreign body entering through orifice.  Our adapted definition further excluded burns (as these are unlikely due to a drug interaction), consistent with work by Sears et al. *J Occup Rehabil* 2015;25(4). |
|  |  | dislocation | 830–839 |  |  |
|  |  | sprain/strain | 840–848 |  |  |
|  |  | intracranial injury | 850–854 |  |  |
|  |  | internal injury of thorax, abdomen, or pelvis | 860–869 |  |  |
|  |  | open wound | 870–897 |  |  |
|  |  | injury to blood vessels | 900–904 |  |  |
|  |  | crushing injury | 925–929 |  |  |
|  |  | injury to nerves or spinal cord | 950–957 |  |  |
|  |  | certain traumatic complications and unspecified injuries | 958–959 |  |  |
| Secondary | Typical hip fracture | closed transcervical fracture | 820.00–820.09 | Principal inpatient discharge diagnosis on an inpatient hospitalization claim | The inclusion of codes for typical closed hip fractures is supported by [FDA Sentinel](https://www.sentinelinitiative.org/sentinel/surveillance-tools/validations-lit-review/health-outcome-algorithm-inventory) and findings by Narogroeknawin et al. *Journal of Clinical Densitometry* 2012;15(1) using 2004–2008 health system data from the University of Alabama at Birmingham. The PPV for typical closed hip fracture ICD-9 discharge diagnosis codes in a principal position was 94%.  The rationale for including typical open hip fractures codes was that such fractures can be caused by an injury.  A hip fracture algorithm including typical open and closed hip fracture codes in 1996–1997 Norwegian data had a PPV and sensitivity of 84% and 90%, respectively (Lofthus et al. *J Clin Epidemiol* 2005;58). Typical open and closed hip fracture codes are a component of [AHRQ’s hip fracture mortality rate quality indicator](https://www.qualityindicators.ahrq.gov/Downloads/Modules/IQI/V50-ICD10/TechSpecs/IQI%2019%20Hip%20Fracture%20Mortality%20Rate.pdf).  The rationale for excluding pathologic fracture codes (e.g., ICD-9 733.14) was that such fractures are due to a localized process such as malignancy or infection (Curtis et al. *Osteoporos Int* 2009;20); their exclusion should have had minimal impact on precision, as ~3% of closed hip fractures are coded as pathologic.  The rationale for excluding atypical (i.e., subtrochanteric and diaphyseal) hip fracture codes (e.g., ICD-9 820.22) was that such fractures are associated with no or minimal trauma and commonly attributed to the use of bisphosphonates and/or glucocorticoids (Shane et al. *JBMR* 2014;29). |
|  |  | open transcervical fracture | 820.10–820.19 |  |  |
|  |  | closed fracture of unspecified trochanteric section of femur | 820.20 |  |  |
|  |  | closed fracture of intertrochanteric section of femur | 820.21 |  |  |
|  |  | open fracture of unspecified trochanteric section of femur | 820.30 |  |  |
|  |  | open fracture of intertrochanteric section of femur | 820.31 |  |  |
|  |  | closed fracture of unspecified part of neck of femur | 820.8 |  |  |
|  |  | open fracture of unspecified part of neck of femur | 820.9 |  |  |
| Secondary | Unintentional motor vehicle crash while subject was driving | MVTA, injuring the driver, in collision with train | E810.0 | Unintentional traumatic injury (see primary above)  and  Any-position discharge diagnosis, arising from the same hospital presentation as the injury, on an ED or inpatient hospitalization claim | The PPV and sensitivity for the motor vehicle crash component of the outcome definition are 88–89% and 97%, respectively ([LeMier et al. *Inj Prev* 2001;7](https://injuryprevention.bmj.com/content/7/4/334.long) and [Bowman et al. *Perspect Health Inf Manag* 2011;8](https://www.ncbi.nlm.nih.gov/pubmed/22016669)).  The inclusion of motor vehicle accident codes of an unintentional manner and exclusion of self-inflicted, assault, and undetermined manner motor vehicle accident codes is supported by the CDC Injury Center’s [E-Code Grouping Matrix](https://www.cdc.gov/injury/wisqars/ecode_matrix.html). |
|  |  | MVTA, injuring the driver, in re-entrant collision with another motor vehicle | E811.0 |  |  |
|  |  | MVTA, injuring the driver, other, in collision with motor vehicle | E812.0 |  |  |
|  |  | MVTA, injuring the driver, in collision with other vehicle | E813.0 |  |  |
|  |  | MVTA, injuring the driver, in collision with pedestrian | E814.0 |  |  |
|  |  | MVTA, injuring the driver, other, in collision on highway | E815.0 |  |  |
|  |  | MVTA, injuring the driver, loss of control, without collision on highway | E816.0 |  |  |
|  |  | MVTA, injuring the driver, noncollision, while boarding or alighting | E817.0 |  |  |
|  |  | MVTA, injuring the driver, other noncollision | E818.0 |  |  |
|  |  | MVTA, injuring the driver, unspecified nature | E819.0 |  |  |
|  |  | MVNTA, injuring the driver, motor-driven snow vehicle | E820.0 |  |  |
|  |  | MVNTA, injuring the driver, off-road motor vehicle | E821.0 |  |  |
|  |  | MVNTA, injuring the driver, other, in collision with moving object | E822.0 |  |  |
|  |  | MVNTA, injuring the driver, other, in collision with stationary object | E823.0 |  |  |
|  |  | MVNTA, injuring the driver, other, while boarding or alighting | E824.0 |  |  |
|  |  | MVNTA, injuring the driver, unspecified nature | E825.0 |  |  |
| AHRQ = Agency for Healthcare Research and Quality; CDC = Centers for Disease Control and Prevention; ED = emergency department; FDA = Food and Drug Administration; ICD-9-CM = international classification of diseases 9^th^ revision clinical modification; MVTA = motor vehicle traffic accident; MVNTA = motor vehicle nontraffic accident; NTDB = National Trauma Data Bank; PPV = positive predictive value | | | | | |

**Supplementary Table S2. Descriptors of persons experiencing typical hip fracture while receiving opioid object drugs**

|  | | **Object drug** | | | | | | | | | | | |
| --- | --- | --- | --- | --- | --- | --- | --- | --- | --- | --- | --- | --- | --- |
|  | | Codeine | Fentanyl | Hydrocod | Hydromor | Levorph | Meperidine | Methadone | Morphine | Oxycodone | Oxymorph | Tapent | Tramadol |
| Persons | | 216 | 128 | 1,142 | 44 | no events | 12 | 20 | 134 | 461 | 8 | 10 | 848 |
| Days of observation, sum | | 6,997 | 8,930 | 71,631 | 1,327 |  | 1,810 | 2,920 | 8,833 | 28,294 | 982 | 201 | 43,600 |
| Days of observation, median (Q1–Q3) per episode | | 12.0 (7.0-23.5) | 36.0 (17.0-58.0) | 17.0 (8.0-37.0) | 13.0 (7.0-21.5) |  | 7.5 (4.5-13.0) | 43.5 (21.0-235.0) | 27.0 (12.0-46.0) | 20.0 (10.0-38.0) | 22.5 (11.0-189.0) | 11.0 (7.0-27.0) | 21.0 (11.0-41.0) |
| Typical hip fracture, sum | | 259 | 153 | 1,317 | 58 |  | 16 | 34 | 160 | 541 | 8 | 10 | 996 |
| *Demographics* | | | | | | | | | | | | | |
| Age, median  (Q1–Q3), years | | 79.0 (74.7-82.7) | 79.6 (75.3-82.3) | 79.0 (72.7-82.6) | 73.1 (59.4-81.0) | no events | 75.2 (66.1-77.9) | 75.6 (67.4-78.8) | 76.1 (63.8-80.2) | 75.6 (64.6-81.0) | 68.0 (56.4-74.8) | 70.6 (67.0-75.8) | 80.5 (74.9-84.2) |
| Sex, sum (%) female | | 156 (72.2) | 93 (72.7) | 805 (70.5) | 27 (61.4) |  | 8 (66.7) | 8 (40.0) | 94 (70.1) | 290 (62.9) | 5 (62.5) | 7 (70.0) | 641 (75.6) |
| Race, sum (%) | African American | 165 (76.4) | 98 (76.6) | 889 (77.8) | 35 (79.5) |  | 9 (75.0) | 15 (75.0) | 113 (84.3) | 355 (77.0) | 7 (87.5) | 7 (70.0) | 640 (75.5) |
|  | Asian | 16 (7.4) | 6 (4.7) | 76 (6.7) | 3 (6.8) |  | 0 (0.0) | 0 (0.0) | 8 (6.0) | 35 (7.6) | 1 (12.5) | 1 (10.0) | 70 (8.3) |
|  | Caucasian | 10 (4.6) | 8 (6.3) | 76 (6.7) | 2 (4.5) |  | 1 (8.3) | 2 (10.0) | 6 (4.5) | 25 (5.4) | 0 (0.0) | 0 (0.0) | 68 (8.0) |
|  | Hispanic | 7 (3.2) | 2 (1.6) | 16 (1.4) | 1 (2.3) |  | 0 (0.0) | 1 (5.0) | 1 (0.7) | 4 (0.9) | 0 (0.0) | 0 (0.0) | 17 (2.0) |
|  | Unknown | 18 (8.3) | 14 (10.9) | 85 (7.4) | 3 (6.8) |  | 2 (16.7) | 2 (10.0) | 6 (4.5) | 42 (9.1) | 0 (0.0) | 2 (20.0) | 53 (6.3) |
| Geographic division, sum (%) | East North Central (IN, IL, MI, OH, WI) | 13 (6.0) | 8 (6.3) | 27 (2.4) | 3 (6.8) |  | 0 (0.0) | 3 (15.0) | 9 (6.7) | 32 (6.9) | 1 (12.5) | 0 (0.0) | 37 (4.4) |
|  | East South Central (AL, KY, MS, TN) | 14 (6.5) | 7 (5.5) | 36 (3.2) | 4 (9.1) |  | 0 (0.0) | 3 (15.0) | 9 (6.7) | 33 (7.2) | 0 (0.0) | 0 (0.0) | 38 (4.5) |
|  | Middle Atlantic (NJ, NY, PA) | 22 (10.2) | 19 (14.8) | 136 (11.9) | 3 (6.8) |  | 0 (0.0) | 1 (5.0) | 19 (14.2) | 54 (11.7) | 2 (25.0) | 1 (10.0) | 101 (11.9) |
|  | Mountain (AZ, CO, ID, NM, MT, UT, NV, WY) | 27 (12.5) | 19 (14.8) | 87 (7.6) | 2 (4.5) |  | 0 (0.0) | 1 (5.0) | 7 (5.2) | 43 (9.3) | 0 (0.0) | 0 (0.0) | 59 (7.0) |
|  | New England  (CT, ME, MA, NH, RI, VT) | 25 (11.6) | 21 (16.4) | 237 (20.8) | 12 (27.3) |  | 5 (41.7) | 4 (20.0) | 34 (25.4) | 130 (28.2) | 1 (12.5) | 4 (40.0) | 205 (24.2) |
|  | Pacific (AK, CA, HI, OR, WA) | 6 (2.8) | 10 (7.8) | 60 (5.3) | 0 (0.0) |  | 0 (0.0) | 0 (0.0) | 7 (5.2) | 8 (1.7) | 0 (0.0) | 0 (0.0) | 34 (4.0) |
|  | South Atlantic (DE, DC, FL, GA, MD, NC, SC, VA, WV) | 22 (10.2) | 10 (7.8) | 147 (12.9) | 4 (9.1) |  | 2 (16.7) | 1 (5.0) | 13 (9.7) | 20 (4.3) | 0 (0.0) | 2 (20.0) | 144 (17.0) |
|  | West North Central (IA, KS, MN, MO, NE, ND, SD) | 22 (10.2) | 5 (3.9) | 133 (11.6) | 2 (4.5) |  | 4 (33.3) | 2 (10.0) | 17 (12.7) | 81 (17.6) | 4 (50.0) | 1 (10.0) | 90 (10.6) |
|  | West South Central (AR, LA, OK, TX) | 62 (28.7) | 29 (22.7) | 266 (23.3) | 13 (29.5) |  | 1 (8.3) | 5 (25.0) | 19 (14.2) | 54 (11.7) | 0 (0.0) | 2 (20.0) | 134 (15.8) |
|  | Unknown | 3 (1.4) | 0 (0.0) | 13 (1.1) | 1 (2.3) |  | 0 (0.0) | 0 (0.0) | 0 (0.0) | 6 (1.3) | 0 (0.0) | 0 (0.0) | 6 (0.7) |
| *Time-varying covariates* | | | | | | | | | | | | | |
| Object drug average daily dose, median (Q1–Q3), MME | | 9.0 (9.0-25.7) | 60.0 (30.0-120) | 20.0 (10.0-30.0) | 128 (50.0-128) | no events | 10.0 (10.0-10.0) | 60.0 (40.0-939) | 60.0 (30.0-120) | 45.0 (25.0-87.3) | 60.0 (60.0-60.0) | 90.0 (80.0-150) | 15.0 (10.0-20.0) |
| Typical hip fracture, ever prior to the day of observation,^a^ person-days (%) | | 2,524 (36.1) | 3,961 (44.4) | 22,610 (31.6) | 478 (36.0) |  | 74 (4.1) | 357 (12.2) | 2,721 (30.8) | 12,247 (43.3) | 67 (6.8) | 75 (37.3) | 16,993 (39.0) |
| hydrocod = hydrocodone; hydromor = hydromorphone; levorph = levorphanol; MME = morphine milligram equivalents; oxymorph = oxymorphone; Q = quartile; tapent = tapentadol  ^a^ diagnosis (any position, any claim type) ever prior to the day of observation | | | | | | | | | | | | | |

**Supplementary Table S3. Descriptors of persons experiencing motor vehicle crash (in which the subject was driving) while receiving opioid object drugs**

|  | | **Object drug** | | | | | | | | | | | |
| --- | --- | --- | --- | --- | --- | --- | --- | --- | --- | --- | --- | --- | --- |
|  | | Codeine | Fentanyl | Hydrocod | Hydromor | Levorph | Meperidine | Methadone | Morphine | Oxycodone | Oxymorph | Tapent | Tramadol |
| Persons | | 92 | 12 | 246 | 9 | no events | 2 | 6 | 16 | 113 | 4 | no events | 98 |
| Days of observation, sum | | 1,154 | 661 | 21,032 | 519 |  | 46 | 463 | 2,305 | 14,534 | 682 |  | 4,956 |
| Days of observation, median (Q1–Q3) per episode | | 9.0 (5.0-16.5) | 37.0 (18.0-74.0) | 15.0 (8.0-39.0) | 19.0 (11.0-71.0) |  | 23.0 (9.0-37.0) | 69.5 (34.0-79.0) | 83.0 (31.5-207.0) | 21.0 (7.0-103.0) | 40.0 (31.5-309.5) |  | 19.0 (10.0-37.0) |
| Motor vehicle crash in which the subject was driving, sum | | 98 | 12 | 262 | 9 |  | 2 | 6 | 16 | 122 | 5 |  | 100 |
| *Demographics* | | | | | | | | | | | | | |
| Age in years, median  (Q1–Q3), years | | 46.8 (32.9-60.6) | 53.2 (43.3-67.7) | 52.5 (38.5-65.6) | 60.2 (43.1-61.8) | no events | 38.1 (26.6-49.6) | 48.7 (43.3-60.2) | 56.4 (46.9-64.3) | 51.2 (39.3-61.2) | 53.6 (46.8-59.2) | no events | 65.2 (48.2-72.3) |
| Sex, sum (%) female | | 58 (63.0) | 7 (58.3) | 129 (52.4) | 4 (44.4) |  | 1 (50.0) | 2 (33.3) | 4 (25.0) | 49 (43.4) | 1 (25.0) |  | 66 (67.3) |
| Race, sum (%) | African American | 42 (45.7) | 8 (66.7) | 142 (57.7) | 8 (88.9) |  | 1 (50.0) | 5 (83.3) | 10 (62.5) | 73 (64.6) | 3 (75.0) |  | 56 (57.1) |
|  | Asian | 16 (17.4) | 2 (16.7) | 30 (12.2) | 0 (0.0) |  | 1 (50.0) | 0 (0.0) | 2 (12.5) | 18 (15.9) | 0 (0.0) |  | 21 (21.4) |
|  | Caucasian | 8 (8.7) | 0 (0.0) | 26 (10.6) | 0 (0.0) |  | 0 (0.0) | 0 (0.0) | 2 (12.5) | 4 (3.5) | 0 (0.0) |  | 9 (9.2) |
|  | Hispanic | 5 (5.4) | 0 (0.0) | 2 (0.8) | 0 (0.0) |  | 0 (0.0) | 0 (0.0) | 0 (0.0) | 1 (0.9) | 0 (0.0) |  | 2 (2.0) |
|  | Unknown | 21 (22.8) | 2 (16.7) | 46 (18.7) | 1 (11.1) |  | 0 (0.0) | 1 (16.7) | 2 (12.5) | 17 (15.0) | 1 (25.0) |  | 10 (10.2) |
| Geographic division, sum (%) | East North Central (IN, IL, MI, OH, WI) | 8 (8.7) | 1 (8.3) | 12 (4.9) | 1 (11.1) |  | 0 (0.0) | 0 (0.0) | 2 (12.5) | 9 (8.0) | 0 (0.0) |  | 3 (3.1) |
|  | East South Central (AL, KY, MS, TN) | 1 (1.1) | 0 (0.0) | 3 (1.2) | 1 (11.1) |  | 0 (0.0) | 0 (0.0) | 0 (0.0) | 3 (2.7) | 0 (0.0) |  | 1 (1.0) |
|  | Middle Atlantic (NJ, NY, PA) | 12 (13.0) | 1 (8.3) | 37 (15.0) | 1 (11.1) |  | 0 (0.0) | 0 (0.0) | 3 (18.8) | 14 (12.4) | 0 (0.0) |  | 14 (14.3) |
|  | Mountain (AZ, CO, ID, NM, MT, UT, NV, WY) | 7 (7.6) | 2 (16.7) | 23 (9.3) | 0 (0.0) |  | 0 (0.0) | 0 (0.0) | 0 (0.0) | 6 (5.3) | 0 (0.0) |  | 10 (10.2) |
|  | New England  (CT, ME, MA, NH, RI, VT) | 30 (32.6) | 6 (50.0) | 63 (25.6) | 4 (44.4) |  | 0 (0.0) | 5 (83.3) | 7 (43.8) | 47 (41.6) | 2 (50.0) |  | 28 (28.6) |
|  | Pacific (AK, CA, HI, OR, WA) | 6 (6.5) | 1 (8.3) | 28 (11.4) | 1 (11.1) |  | 2 (100.0) | 0 (0.0) | 0 (0.0) | 11 (9.7) | 1 (25.0) |  | 13 (13.3) |
|  | South Atlantic (DE, DC, FL, GA, MD, NC, SC, VA, WV) | 5 (5.4) | 0 (0.0) | 31 (12.6) | 1 (11.1) |  | 0 (0.0) | 1 (16.7) | 0 (0.0) | 7 (6.2) | 0 (0.0) |  | 10 (10.2) |
|  | West North Central (IA, KS, MN, MO, NE, ND, SD) | 11 (12.0) | 1 (8.3) | 24 (9.8) | 0 (0.0) |  | 0 (0.0) | 0 (0.0) | 3 (18.8) | 12 (10.6) | 1 (25.0) |  | 10 (10.2) |
|  | West South Central (AR, LA, OK, TX) | 12 (13.0) | 0 (0.0) | 23 (9.3) | 0 (0.0) |  | 0 (0.0) | 0 (0.0) | 1 (6.3) | 4 (3.5) | 0 (0.0) |  | 9 (9.2) |
|  | Unknown | 0 (0.0) | 0 (0.0) | 2 (0.8) | 0 (0.0) |  | 0 (0.0) | 0 (0.0) | 0 (0.0) | 0 (0.0) | 0 (0.0) |  | 0 (0.0) |
| *Time-varying covariates* | | | | | | | | | | | | | |
| Object drug average daily dose, median (Q1–Q3), MME | | 10.8 (6.0-18.0) | 120 (60.0-180) | 22.5 (15.0-40.0) | 64.0 (48.0-64.0) | no events | 20.0 (20.0-20.0) | 320 (320-320) | 90.0 (30.0-120) | 90.0 (56.3-180) | 90.0 (90.0-90.0) | no events | 10.0 (10.0-18.8) |
| Motor vehicle crash in which subject was driving, ever prior to the day of observation,^a^ person-days (%) | | 357 (30.9) | 188 (28.4) | 8,293 (39.4) | 188 (36.2) |  | 7 (15.2) | 301 (65.0) | 426 (18.5) | 8,406 (57.8) | 556 (81.5) |  | 1,376 (27.8) |
| hydrocod = hydrocodone; hydromor = hydromorphone; levorph = levorphanol; MME = morphine milligram equivalents; oxymorph = oxymorphone; Q = quartile; tapent = tapentadol  ^a^ diagnosis (any position, any claim type) ever prior to the day of observation | | | | | | | | | | | | | |

**Supplementary Table S4. Summary data on rate ratios for typical hip fracture, by object drug**

|  | **Object drug** | | | | | | | | | | | |
| --- | --- | --- | --- | --- | --- | --- | --- | --- | --- | --- | --- | --- |
|  | Codeine | Fentanyl | Hydrocod | Hydromor | Levorph | Meperidine | Methadone | Morphine | Oxycodone | Oxymorph | Tapentadol | Tramadol |
| *Unadjusted* analyses, *before* semi-Bayes shrinkage | | | | | | | | | | | | |
| Drug triplets examined, sum | 42 | 34 | 910 | NA | NA | NA | NA | 36 | 256 | NA | NA | 655 |
| 3DIs, sum (%) | 1 (2.4) | 2 (5.9) | 72 (7.9) | NA | NA | NA | NA | 3 (8.3) | 15 (5.9) | NA | NA | 27 (4.1) |
| Increased rate^a^ | 0 (0.0) | 0 (0.0) | 2 (0.2) | NA | NA | NA | NA | 1 (2.8) | 4 (1.6) | NA | NA | 2 (0.3) |
| Decreased rate^b^ | 1 (2.4) | 2 (5.9) | 70 (7.7) | NA | NA | NA | NA | 2 (5.6) | 11 (4.3) | NA | NA | 25 (3.8) |
| RR geometric mean ± SD | 0.69 ± 3.08 | 0.52 ± 2.66 | 0.58 ± 3.03 | NA | NA | NA | NA | 0.85 ± 3.47 | 0.63 ± 3.05 | NA | NA | 0.64 ± 3.02 |
| RR range, min to max | 0.05 - 8.12 | 0.03 - 5.33 | 0.02 - 39.50 | NA | NA | NA | NA | 0.07 - 14.70 | 0.05 - 26.19 | NA | NA | 0.03 - 19.30 |
| *Confounder-adjusted* analyses, *before* semi-Bayes shrinkage | | | | | | | | | | | | |
| Drug triplets examined, sum | NA | 5 | 647 | NA | NA | NA | NA | NA | 160 | NA | NA | 238 |
| 3DIs, sum (%) | NA | 1 (20.0) | 50 (7.7) | NA | NA | NA | NA | NA | 6 (3.8) | NA | NA | 11 (4.6) |
| Increased rate^a^ | NA | 0 (0.0) | 4 (0.6) | NA | NA | NA | NA | NA | 2 (1.3) | NA | NA | 0 (0.0) |
| Decreased rate^b^ | NA | 1 (20.0) | 46 (7.1) | NA | NA | NA | NA | NA | 4 (2.5) | NA | NA | 11 (4.6) |
| RR geometric mean ± SD | NA | 0.56 ± 2.80 | 0.63 ± 3.49 | NA | NA | NA | NA | NA | 0.74 ± 3.30 | NA | NA | 0.74 ± 2.91 |
| RR range, min to max | NA | 0.13 - 1.47 | 0.01 - 25.28 | NA | NA | NA | NA | NA | 0.05 - 29.75 | NA | NA | 0.03 - 24.50 |
| *Unadjusted* analyses, *after* semi-Bayes shrinkage | | | | | | | | | | | | |
| Drug triplets examined, sum | 42 | 34 | 910 | NA | NA | NA | NA | 36 | 256 | NA | NA | 655 |
| 3DIs, sum (%) | 0 (0.0) | 0 (0.0) | 55 (6.0) | NA | NA | NA | NA | 0 (0.0) | 0 (0.0) | NA | NA | 3 (0.5) |
| Increased rate^a^ | 0 (0.0) | 0 (0.0) | 0 (0.0) | NA | NA | NA | NA | 0 (0.0) | 0 (0.0) | NA | NA | 0 (0.0) |
| Decreased rate^b^ | 0 (0.0) | 0 (0.0) | 55 (6.0) | NA | NA | NA | NA | 0 (0.0) | 0 (0.0) | NA | NA | 3 (0.5) |
| RR geometric mean ± SD | 0.74 ± 1.12 | 0.53 ± 1.18 | 0.56 ± 1.21 | NA | NA | NA | NA | 0.84 ± 1.20 | 0.63 ± 1.20 | NA | NA | 0.68 ± 1.19 |
| RR range, min to max | 0.55 - 0.98 | 0.34 - 0.73 | 0.24 - 1.24 | NA | NA | NA | NA | 0.58 - 1.53 | 0.42 - 1.22 | NA | NA | 0.41 - 1.23 |
| *Confounder-adjusted* analyses, *after* semi-Bayes shrinkage | | | | | | | | | | | | |
| Drug triplets examined, sum | NA | 5 | 647 | NA | NA | NA | NA | NA | 160 | NA | NA | 238 |
| 3DIs, sum (%) | NA | 0 (0.0) | 30 (4.6) | NA | NA | NA | NA | NA | 0 (0.0) | NA | NA | 0 (0.0) |
| Increased rate^a^ | NA | 0 (0.0) | 0 (0.0) | NA | NA | NA | NA | NA | 0 (0.0) | NA | NA | 0 (0.0) |
| Decreased rate^b^ | NA | 0 (0.0) | 30 (4.6) | NA | NA | NA | NA | NA | 0 (0.0) | NA | NA | 0 (0.0) |
| RR geometric mean ± SD | NA | 0.54 ± 1.20 | 0.59 ± 1.23 | NA | NA | NA | NA | NA | 0.71 ± 1.18 | NA | NA | 0.73 ± 1.19 |
| RR range, min to max | NA | 0.40 - 0.64 | 0.22 - 1.37 | NA | NA | NA | NA | NA | 0.49 - 1.23 | NA | NA | 0.43 - 1.26 |
| hydrocod = hydrocodone; hydromor = hydromorphone; levorph = levorphanol; max = maximum; min = minimum; NA = not applicable; oxymorph = oxymorphone; RR = rate ratio; SD = standard deviation; 3DI = drug-drug-drug interaction  ^a^ lower bound of the 95% confidence interval for the RR of interest excluded the null value ^b^ upper bound of the 95% confidence interval for the RR of interest excluded the null value | | | | | | | | | | | | |

**Supplementary Table S5. Summary data on rate ratios for motor vehicle crash (in which the subject was driving), by object drug**

|  | **Object drug** | | | | | | | | | | | |
| --- | --- | --- | --- | --- | --- | --- | --- | --- | --- | --- | --- | --- |
|  | Codeine | Fentanyl | Hydrocod | Hydromor | Levorph | Meperidine | Methadone | Morphine | Oxycodone | Oxymorph. | Tapentadol | Tramadol |
| *Unadjusted* analyses, *before* semi-Bayes shrinkage | | | | | | | | | | | | |
| Drug triplets examined, sum | 2 | NA | 11 | NA | NA | NA | NA | NA | 5 | NA | NA | NA |
| 3DIs, sum (%) | 0 (0.0) | NA | 1 (9.1) | NA | NA | NA | NA | NA | 0 (0.0) | NA | NA | NA |
| Increased rate^a^ | 0 (0.0) | NA | 0 (0.0) | NA | NA | NA | NA | NA | 0 (0.0) | NA | NA | NA |
| Decreased rate^b^ | 0 (0.0) | NA | 1 (9.1) | NA | NA | NA | NA | NA | 0 (0.0) | NA | NA | NA |
| RR geometric mean ± SD | 1.01 ± 1.13 | NA | 0.32 ± 3.46 | NA | NA | NA | NA | NA | 0.99 ± 2.49 | NA | NA | NA |
| RR range, min to max | 0.92 - 1.10 | NA | 0.04 - 1.86 | NA | NA | NA | NA | NA | 0.25 - 2.30 | NA | NA | NA |
| *Unadjusted* analyses, *after* semi-Bayes shrinkage | | | | | | | | | | | | |
| Drug triplets examined, sum | 2 | NA | 11 | NA | NA | NA | NA | NA | 5 | NA | NA | NA |
| 3DIs, sum (%) | 0 (0.0) | NA | 5 (45.5) | NA | NA | NA | NA | NA | 0 (0.0) | NA | NA | NA |
| Increased rate^a^ | 0 (0.0) | NA | 0 (0.0) | NA | NA | NA | NA | NA | 0 (0.0) | NA | NA | NA |
| Decreased rate^b^ | 0 (0.0) | NA | 5 (45.5) | NA | NA | NA | NA | NA | 0 (0.0) | NA | NA | NA |
| RR geometric mean ± SD | 1.04 ± 1.01 | NA | 0.37 ± 1.16 | NA | NA | NA | NA | NA | 1.38 ± 1.07 | NA | NA | NA |
| RR range, min to max | 1.03 - 1.05 | NA | 0.30 - 0.51 | NA | NA | NA | NA | NA | 1.28 - 1.49 | NA | NA | NA |
| hydrocod = hydrocodone; hydromor = hydromorphone; levorph = levorphanol; max = maximum; min = minimum; NA = not applicable; oxymorph = oxymorphone; RR = rate ratio; SD = standard deviation; 3DI = drug-drug-drug interaction  ^a^ lower bound of the 95% confidence interval for the RR of interest excluded the null value ^b^ upper bound of the 95% confidence interval for the RR of interest excluded the null value | | | | | | | | | | | | |

**Supplementary Table S6.** **Drug-drug-drug interaction signals with statistically significantly increased rates of unintentional traumatic injury for commonly used opioids, by therapeutic category of base pair precipitant drug | Secondary analysis removing episodes with unintentional traumatic injury prior to the first day of observation**

| **Object** | **Base precipitant, therapeutic category** | **Base precipitant, drug** | **Candidate interacting precipitant, drug** | **Rate ratio, semi-Bayes shrunk and adjusted** | **95% confidence interval** |
| --- | --- | --- | --- | --- | --- |
| HYDROCODONE | Anti-infective | amoxicillin | ibuprofen | 2.12 | 1.04-4.36 |
|  | Cardiovascular | amlodipine | cyclobenzaprine | 2.20 | 1.27-3.80 |
|  |  | amlodipine | gabapentin | 1.98 | 1.04-3.77 |
|  |  | atenolol | gabapentin | 2.16 | 1.09-4.30 |
|  |  | atorvastatin | cephalexin | 1.77 | 1.01-3.10 |
|  |  | diltiazem | acetaminophen | 2.30 | 1.08-4.92 |
|  |  | lisinopril | naproxen | 1.90 | 1.01-3.59 |
|  |  | valsartan | cephalexin | 2.65 | 1.35-5.18 |
|  | Central nervous system | alprazolam | diazepam | 2.31 | 1.01-5.24 |
|  |  | cyclobenzaprine | diazepam | 1.87 | 1.05-3.35 |
|  |  | cyclobenzaprine | metaxalone | 2.00 | 1.01-3.98 |
|  |  | cyclobenzaprine | naproxen | 2.07 | 1.17-3.64 |
|  |  | cyclobenzaprine | prednisone | 1.57 | 1.05-2.34 |
|  |  | gabapentin | cephalexin | 1.79 | 1.01-3.16 |
|  |  | gabapentin | cyclobenzaprine | 1.84 | 1.06-3.21 |
|  |  | gabapentin | sulfamethoxazole | 1.99 | 1.02-3.87 |
|  |  | gabapentin | trimethoprim | 1.95 | 1.01-3.75 |
|  |  | naproxen | ibuprofen | 1.91 | 1.09-3.35 |
|  | Endocrine and metabolic | levothyroxine | cyclobenzaprine | 2.49 | 1.57-3.93 |
|  |  | levothyroxine | gabapentin | 2.04 | 1.21-3.43 |
|  |  | levothyroxine | sulfamethoxazole | 1.90 | 1.01-3.56 |
|  |  | levothyroxine | trimethoprim | 1.93 | 1.03-3.61 |
|  |  | metformin | ibuprofen | 2.28 | 1.17-4.43 |
|  |  | metformin | sulfamethoxazole | 2.27 | 1.19-4.31 |
|  |  | metformin | trimethoprim | 2.27 | 1.19-4.31 |
|  | Gastrointestinal | omeprazole | cyclobenzaprine | 2.00 | 1.09-3.65 |
|  |  | pantoprazole | gabapentin | 2.52 | 1.18-5.39 |
|  | Nutrients and nutritional | potassium chloride | gabapentin | 1.95 | 1.08-3.51 |
|  |  | potassium chloride | niacin | 2.19 | 1.04-4.61 |
|  | Renal and genitourinary | furosemide | gabapentin | 2.21 | 1.28-3.79 |
|  |  | furosemide | sulfamethoxazole | 2.42 | 1.41-4.18 |
|  |  | furosemide | trimethoprim | 2.42 | 1.41-4.18 |
|  |  | hydrochlorothiazide | cephalexin | 1.79 | 1.13-2.84 |
| OXYCODONE | Cardiovascular | amlodipine | ibuprofen | 2.41 | 1.18-4.93 |
|  |  | lisinopril | cyclobenzaprine | 2.11 | 1.15-3.87 |
|  |  | metoprolol | cephalexin | 1.98 | 1.01-3.86 |
|  |  | simvastatin | cephalexin | 2.16 | 1.11-4.22 |
|  | Endocrine and metabolic | levothyroxine | buspirone | 2.29 | 1.09-4.81 |
|  |  | levothyroxine | ciprofloxacin | 2.25 | 1.23-4.12 |
|  |  | levothyroxine | ibuprofen | 2.43 | 1.06-5.60 |
|  |  | metformin | ibuprofen | 2.63 | 1.12-6.20 |
|  | Gastrointestinal | omeprazole | ciprofloxacin | 2.10 | 1.12-3.93 |
|  | Renal and genitourinary | furosemide | fluconazole | 2.42 | 1.10-5.31 |
|  |  | furosemide | ibuprofen | 2.50 | 1.16-5.37 |
| TRAMADOL | Cardiovascular | amlodipine | amoxicillin | 2.00 | 1.02-3.93 |
|  |  | amlodipine | nitrofurantoin | 2.13 | 1.06-4.29 |
|  |  | metoprolol | cephalexin | 2.98 | 1.59-5.58 |
|  |  | simvastatin | cephalexin | 2.22 | 1.14-4.34 |
|  | Endocrine and metabolic | levothyroxine | cephalexin | 2.06 | 1.14-3.73 |
|  | Gastrointestinal | pantoprazole | cephalexin | 3.59 | 1.57-8.21 |
|  | Hematological | clopidogrel | cephalexin | 2.74 | 1.20-6.22 |
|  | Nutrients and nutritional | potassium chloride | acetaminophen | 2.20 | 1.02-4.73 |
|  |  | potassium chloride | cephalexin | 2.21 | 1.10-4.47 |
|  | Renal and genitourinary | furosemide | acetaminophen | 2.00 | 1.03-3.91 |
|  |  | furosemide | cephalexin | 3.02 | 1.63-5.62 |

**Supplementary Figure S1. Volcano plot depicting opioid + precipitant base pair with candidate interacting precipitant associations with unintentional traumatic injury.**

**
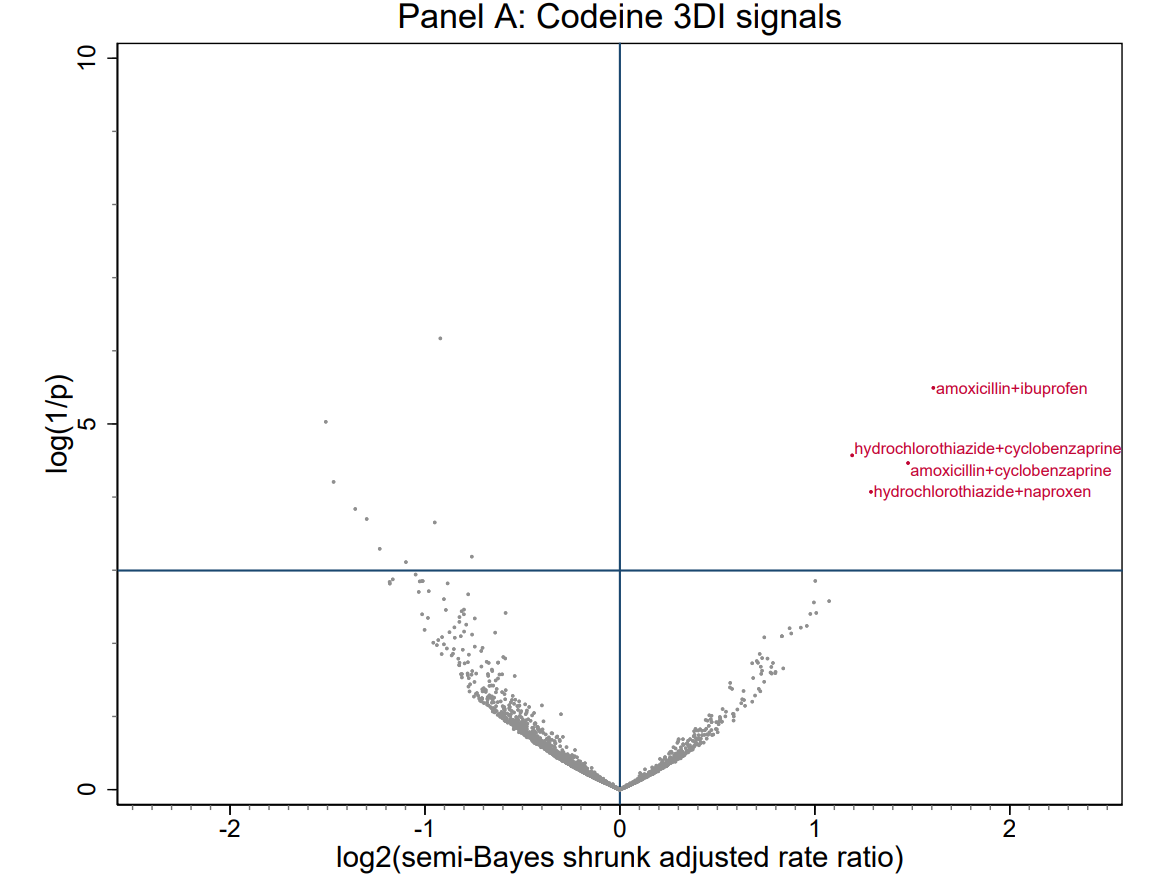
**

**
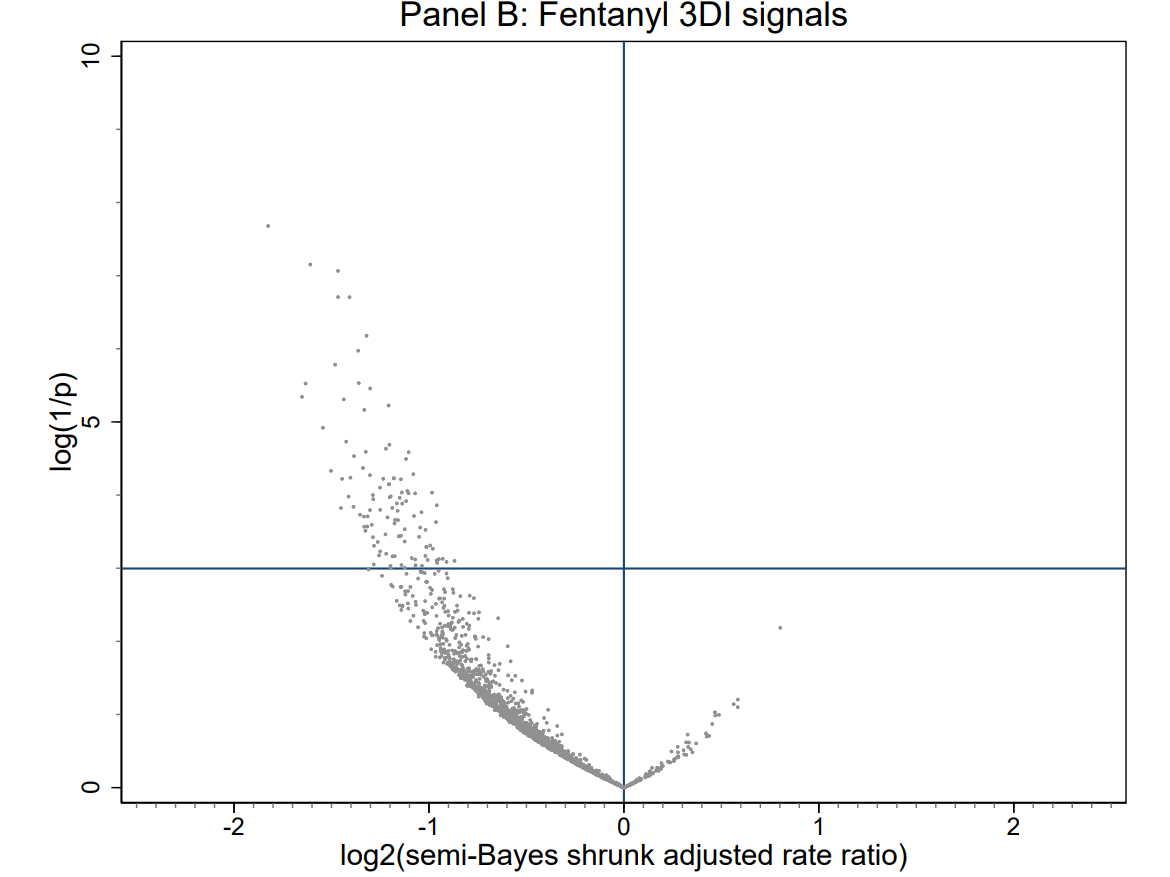
**

**
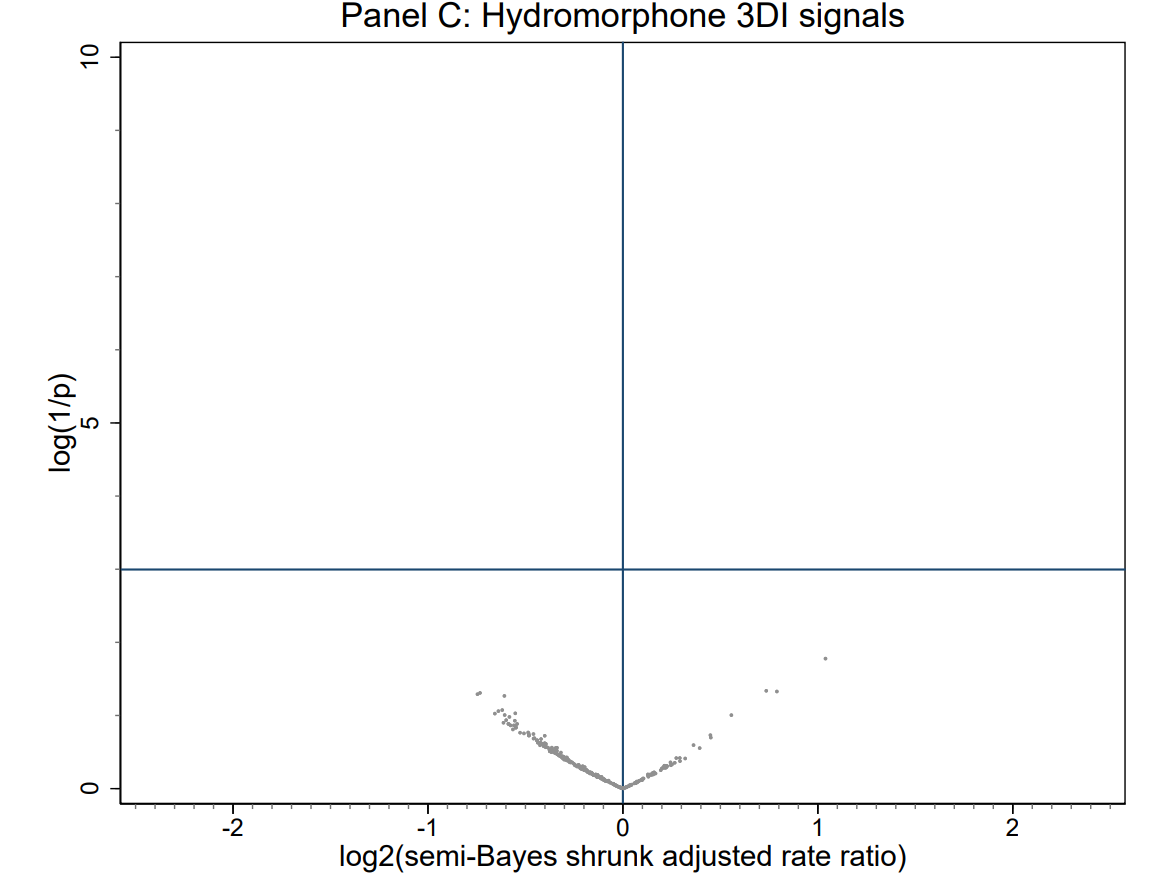
**

**
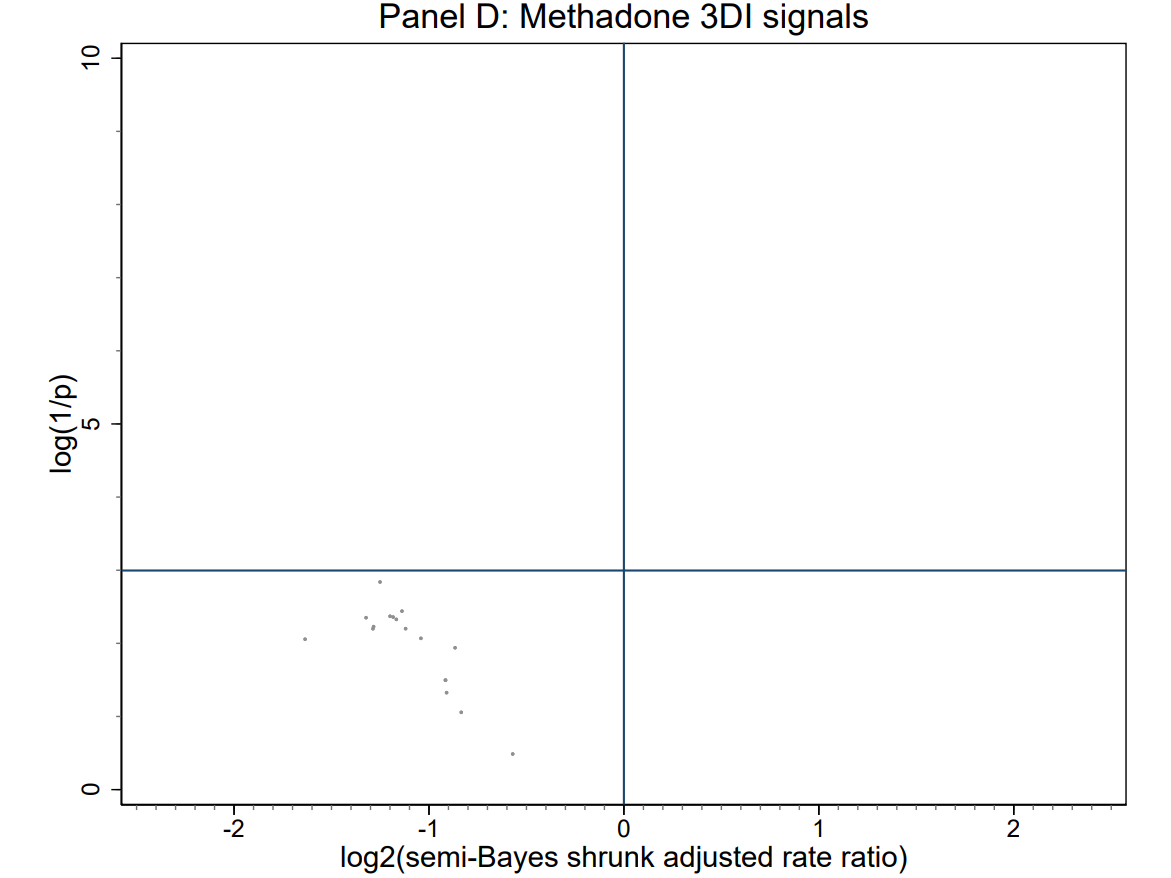
**

**
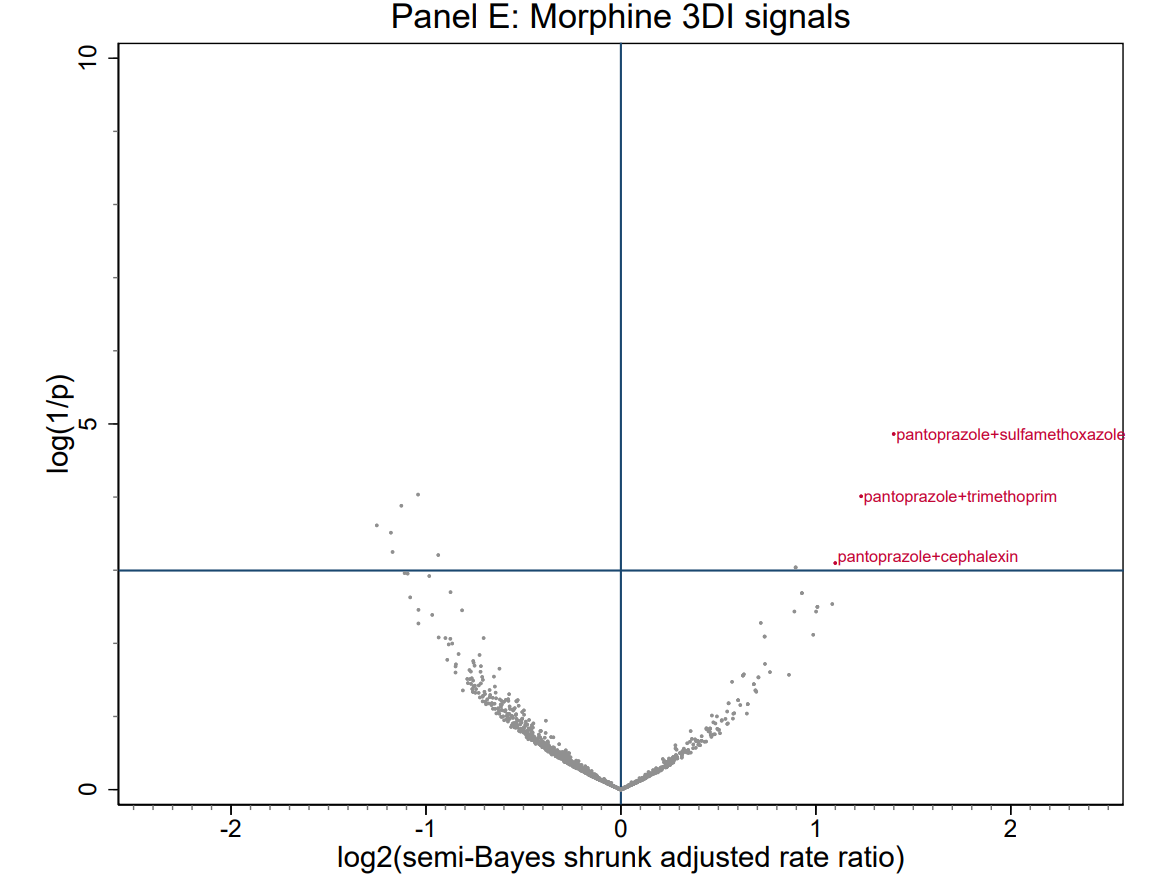
**

**
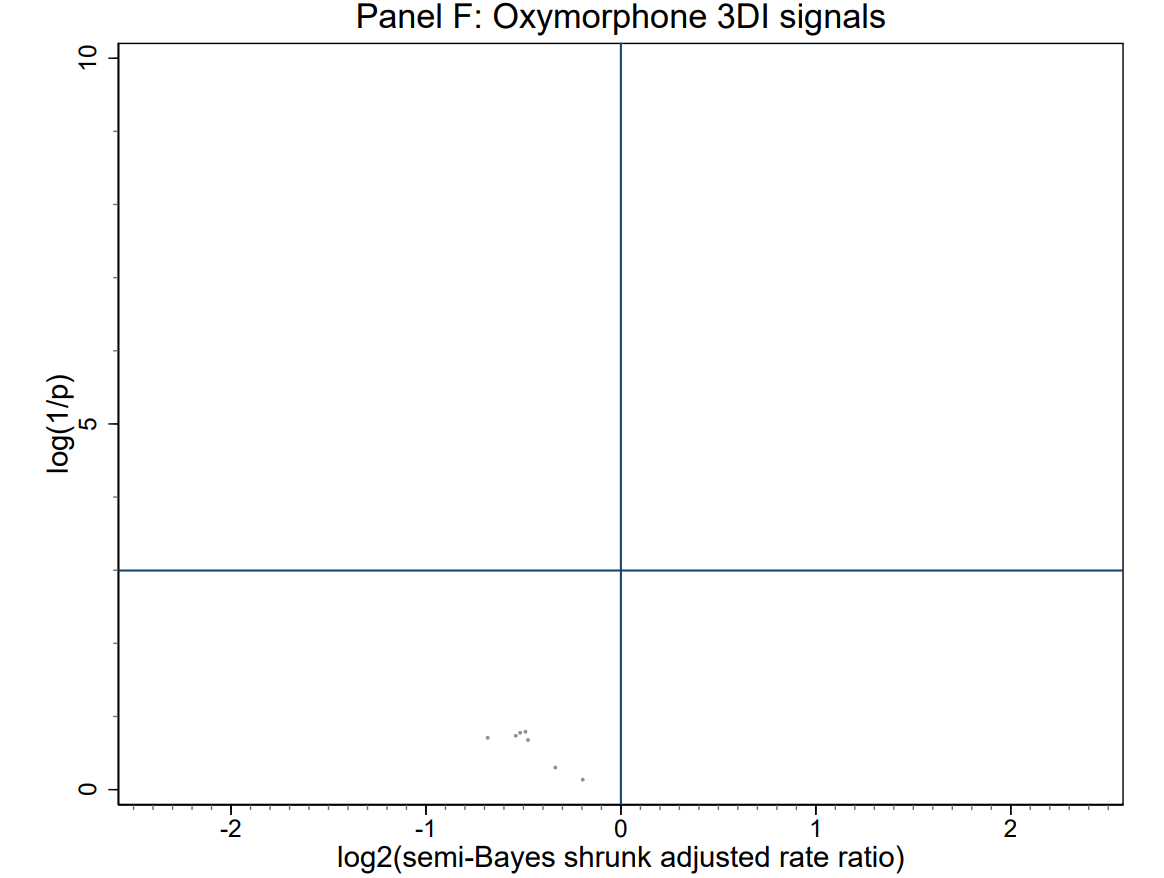
**

Panel A depicts associations with codeine. Panel B depicts associations with fentanyl. Panel C depicts associations with hydromorphone. Panel D depicts associations with methadone. Panel E depicts associations with morphine. Panel F depicts associations with oxymorphone. Semi-Bayes shrinkage prespecified a variance of 0.25, assuming that 95% of true rate ratios would fall within an unspecified 7-fold range of each other. The x-axis represents the log base 2 semi-Bayes shrunk adjusted rate ratio for opioid + precipitant base pair with candidate interacting precipitant vs. opioid + precipitant base pair. The y-axis represents the log (1 / p-value) for the semi-Bayes shrunk adjusted rate ratio. Data points in the upper right quadrant represent drug triplets with a statistically significant signal for elevated risk of unintentional traumatic injury. For ease of reading, we limited labeling to upper right quadrant data points with log base 2 semi-Bayes shrunk adjusted rate ratio ≥1 or log (1 / p-value) ≥10. We excluded signals with propoxyphene (a medical product eventually withdrawn from the United States market) from the plots, as they may have represented opioid switching rather than concomitant therapy. 3DI = drug-drug-drug interactions.

**Supplementary Figure S2. Volcano plot depicting opioid + precipitant base pair with candidate interacting precipitant associations with** **typical hip fracture.**


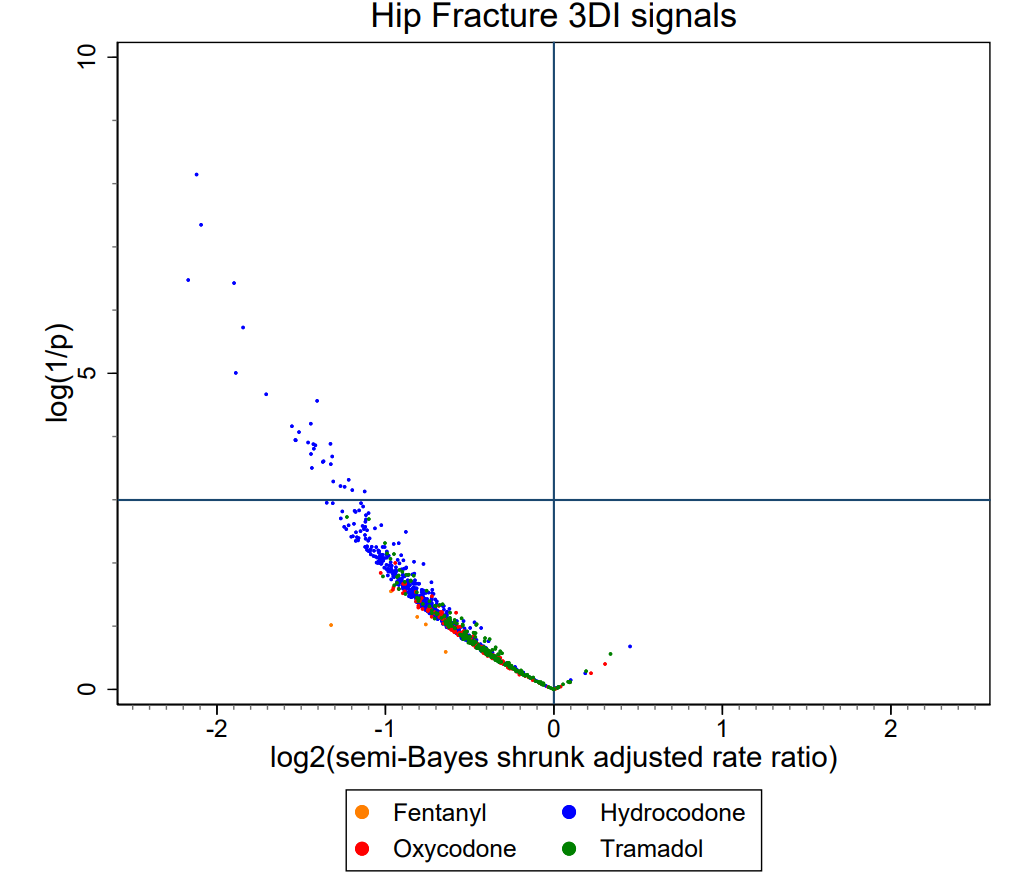


Semi-Bayes shrinkage prespecified a variance of 0.25, assuming that 95% of true rate ratios would fall within an unspecified 7-fold range of each other. The x-axis represents the log base 2 semi-Bayes shrunk adjusted rate ratio for opioid + precipitant base pair with candidate interacting precipitant vs. opioid + precipitant base pair. The y-axis represents the log (1 / p-value) for the semi-Bayes shrunk adjusted rate ratio. Data points in the upper right quadrant represent drug triplets with a statistically significant signal for elevated risk of typical hip fracture. We excluded signals with propoxyphene (a medical product eventually withdrawn from the United States market) from the plots, as they may have represented opioid switching rather than concomitant therapy. 3DI = drug-drug-drug interactions.

**Supplementary Figure S3. Volcano plot depicting opioid + precipitant base pair with candidate interacting precipitant associations with unintentional traumatic injury | Prespecified secondary analysis increasing semi-Bayes shrinkage variance parameter.**

**
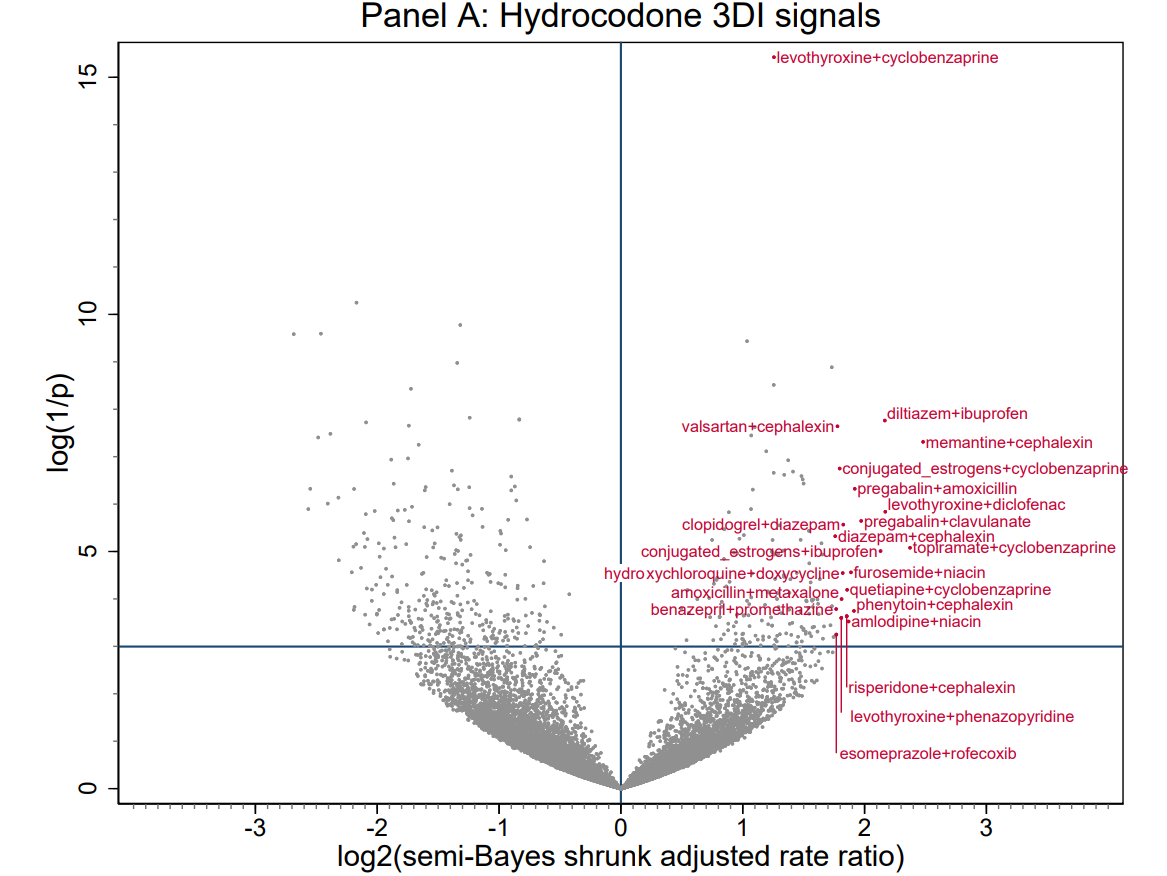
**

**
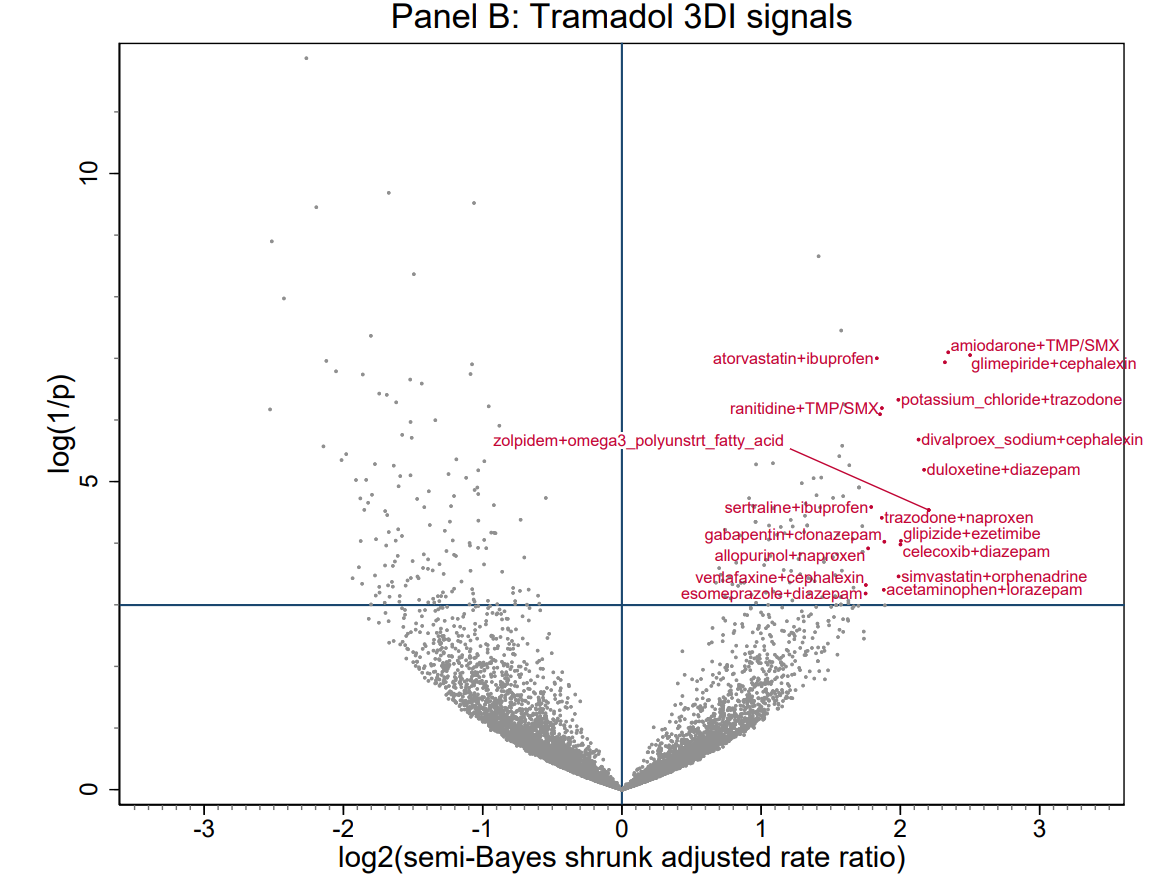
**

**
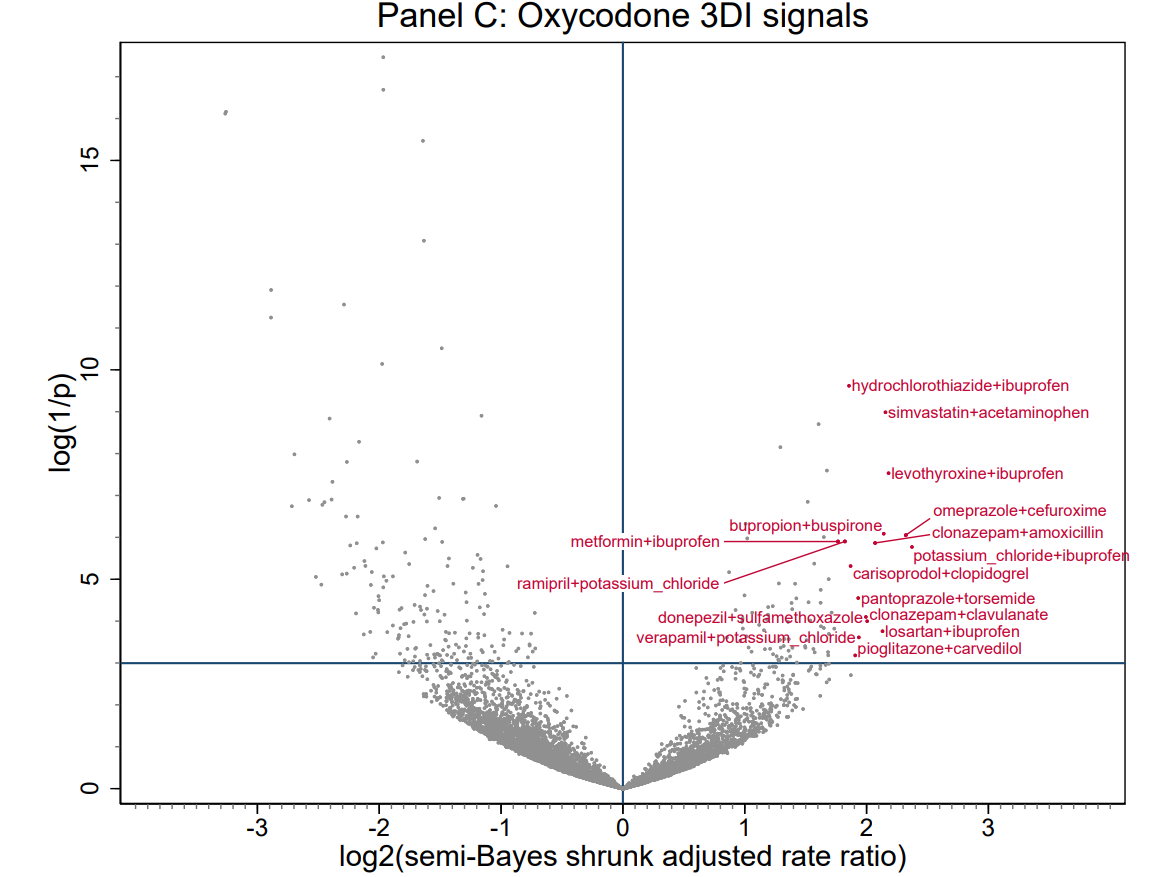
**

**
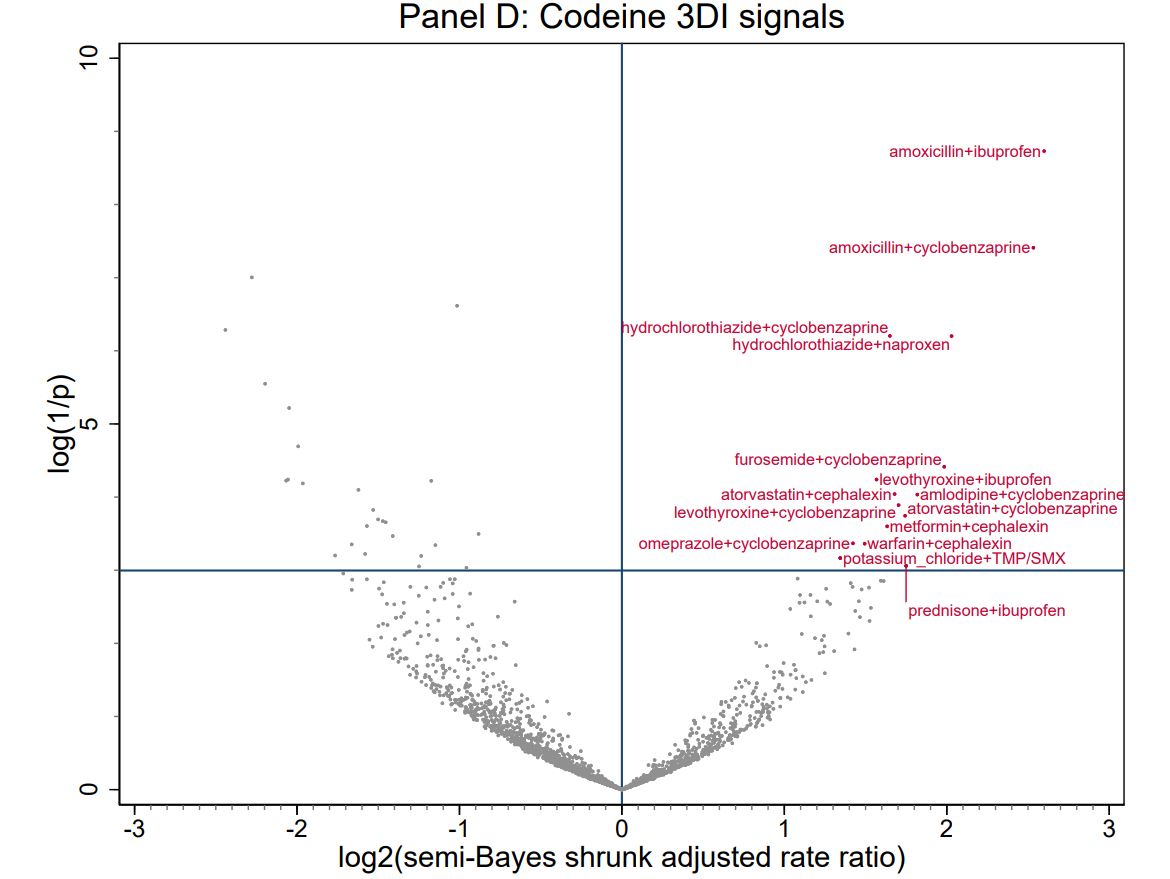
**

**
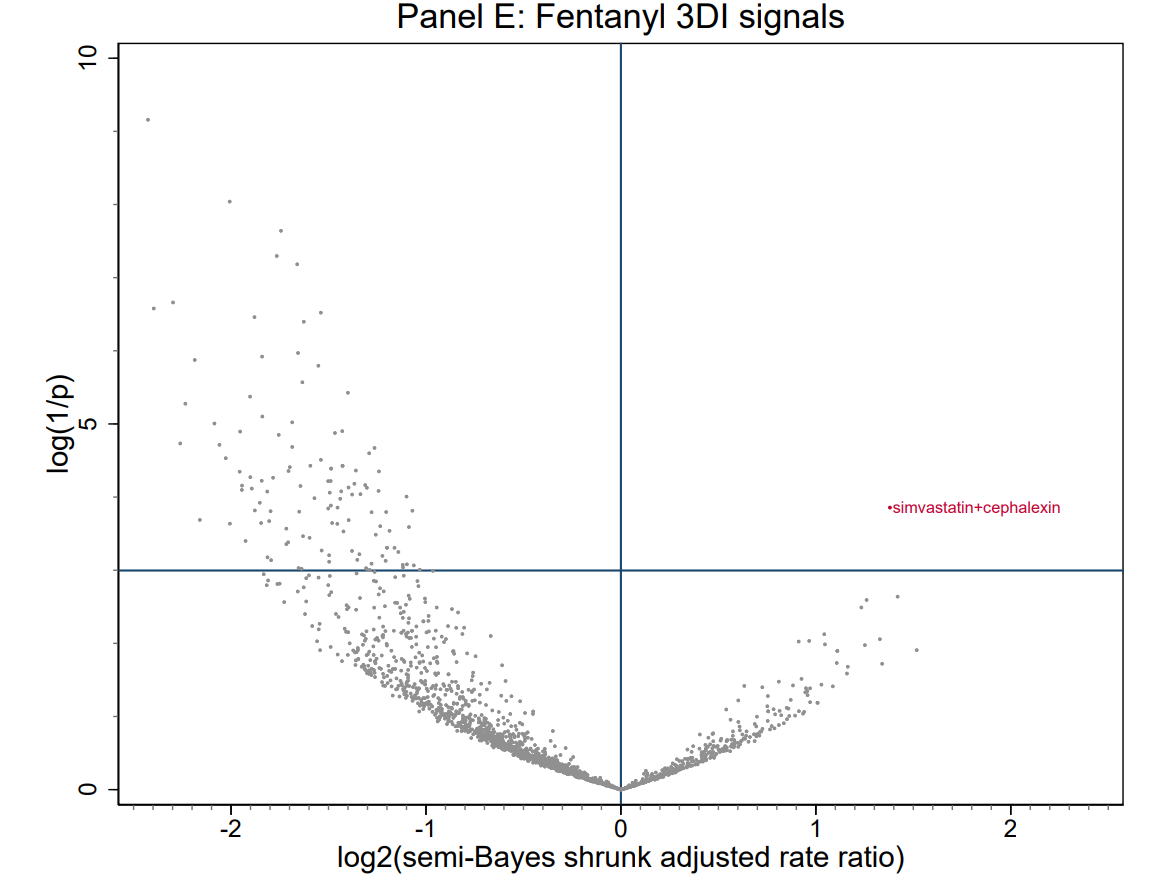
**

**
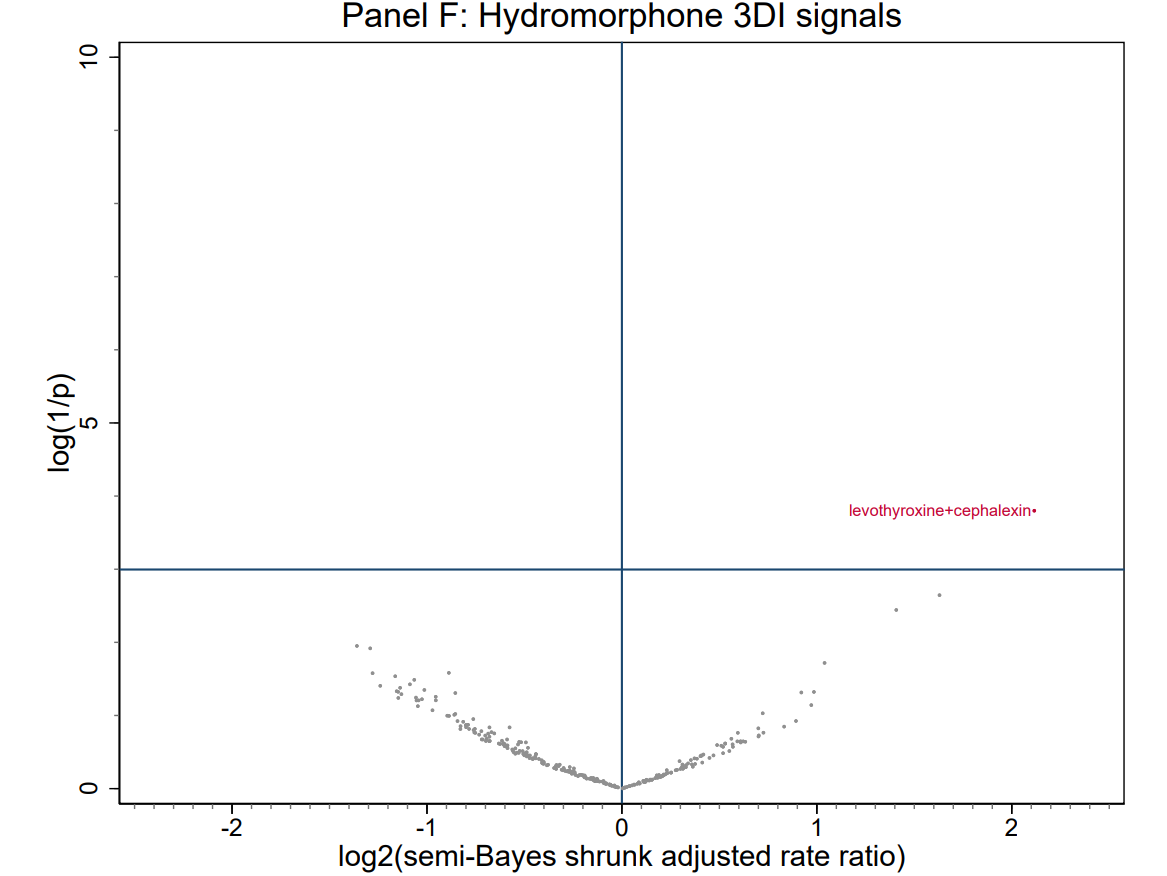
**

**
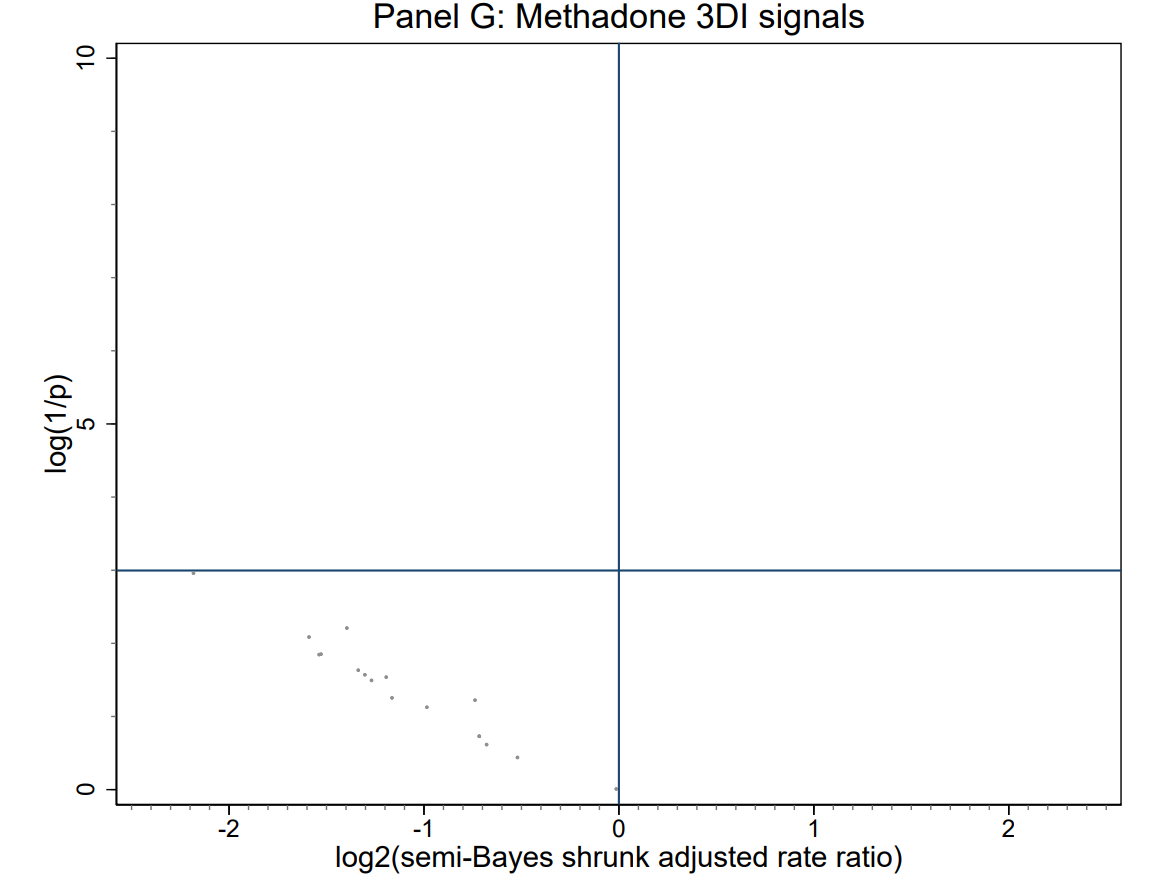
**

**
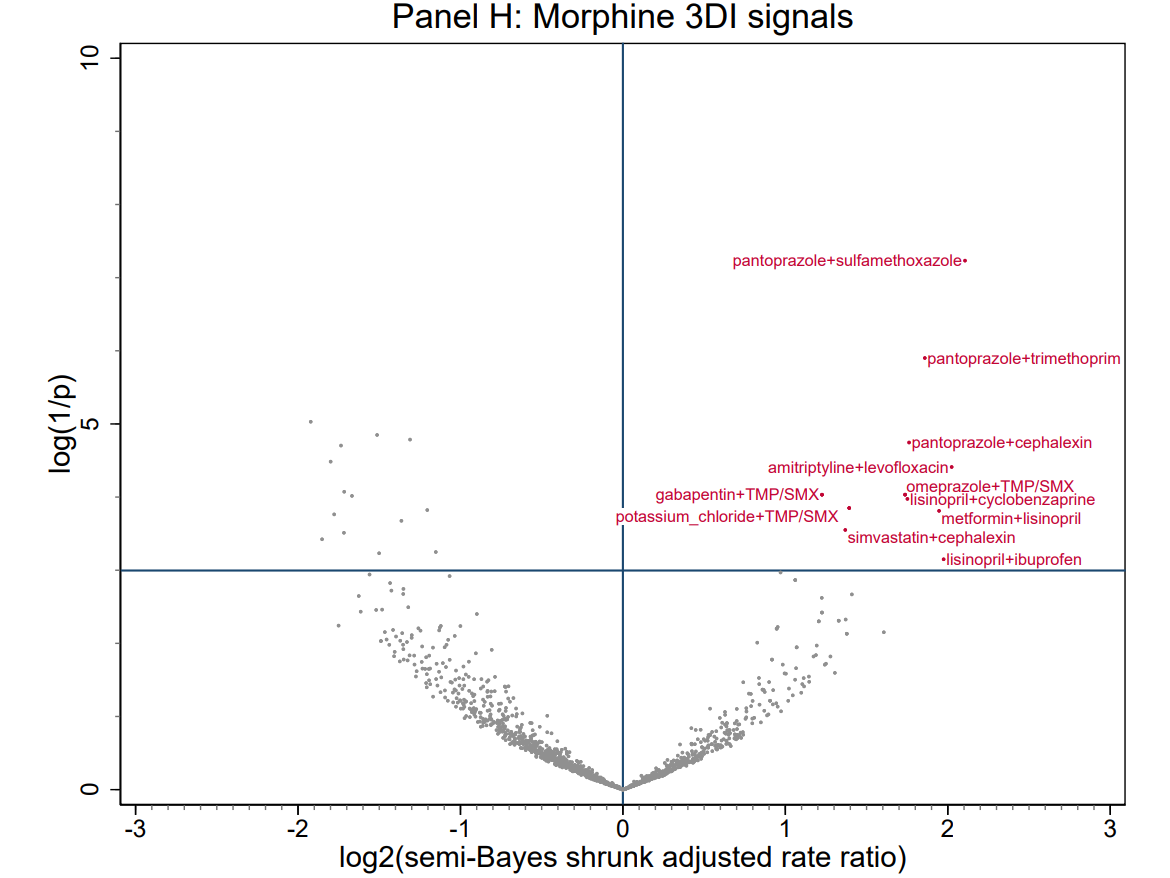
**

**
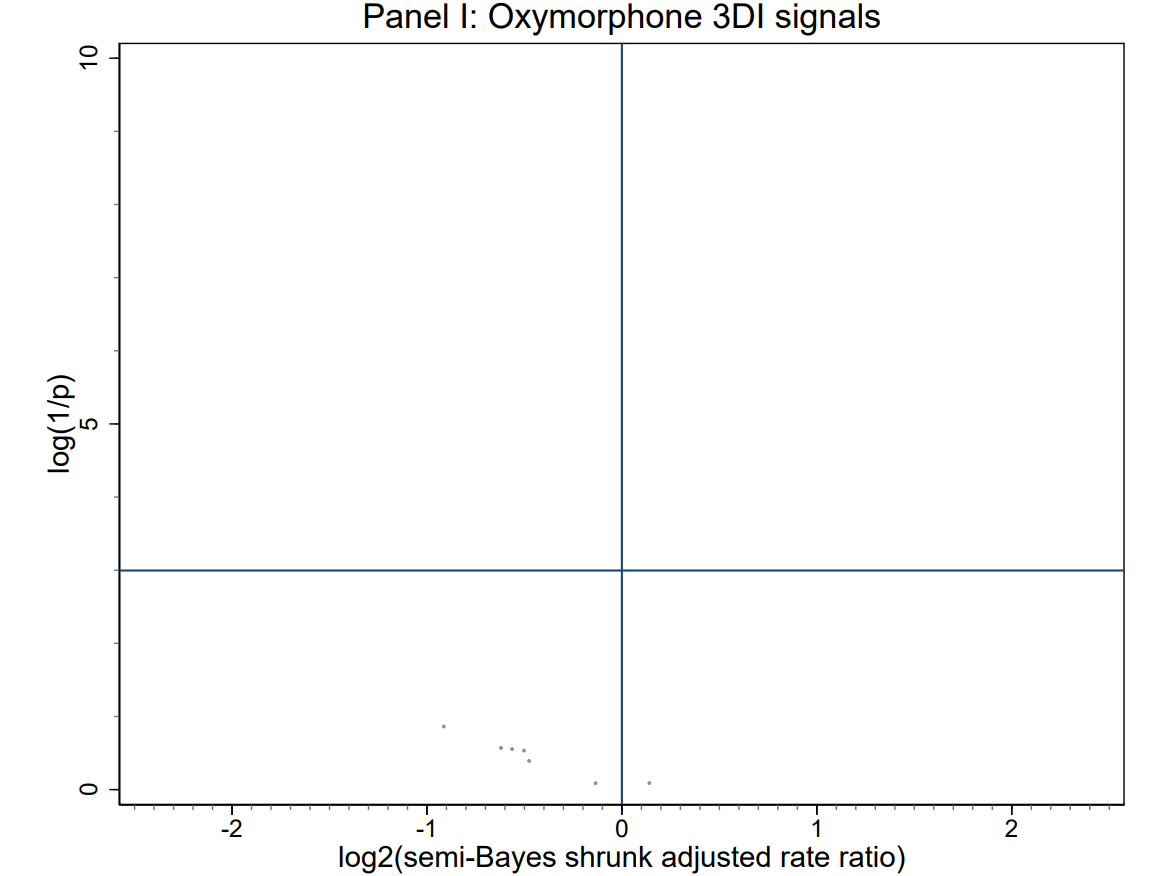
**

Panel A depicts associations with hydrocodone. Panel B depicts associations with tramadol. Panel C depicts associations with oxycodone. Panel D depicts associations with codeine. Panel E depicts associations with fentanyl. Panel F depicts associations with hydromorphone. Panel G depicts associations with methadone. Panel H depicts associations with morphine. Panel I depicts associations with oxymorphone. Semi-Bayes shrinkage prespecified a variance of 0.67, assuming that 95% of true rate ratios would fall within an unspecified 25-fold range of each other. The x-axis represents the log base 2 semi-Bayes shrunk adjusted rate ratio for opioid + precipitant base pair with candidate interacting precipitant vs. opioid + precipitant base pair. The y-axis represents the log (1 / p-value) for the semi-Bayes shrunk adjusted rate ratio. Data points in the upper right quadrant represent drug triplets with a statistically significant signal for elevated risk of unintentional traumatic injury. For ease of reading for Panels A-C, we limited labeling to upper right quadrant data points with log base 2 semi-Bayes shrunk adjusted rate ratio ≥ 1.75 or log (1 / p-value) ≥10. For ease of reading for Panels D-I, we limited labeling to upper right quadrant data points with log base 2 semi-Bayes shrunk adjusted rate ratio ≥1 or log (1 / p-value) ≥10. We excluded signals with propoxyphene (a medical product eventually withdrawn from the United States market) from the plots, as they may have represented opioid switching rather than concomitant therapy. 3DI = drug-drug-drug interactions, SMX = sulfamethoxazole, TMP = trimethoprim.

**Supplementary Figure S4. Volcano plot depicting opioid + precipitant base pair with candidate interacting precipitant associations with** **typical hip fracture | Prespecified secondary analysis increasing semi-Bayes shrinkage variance parameter.**

**
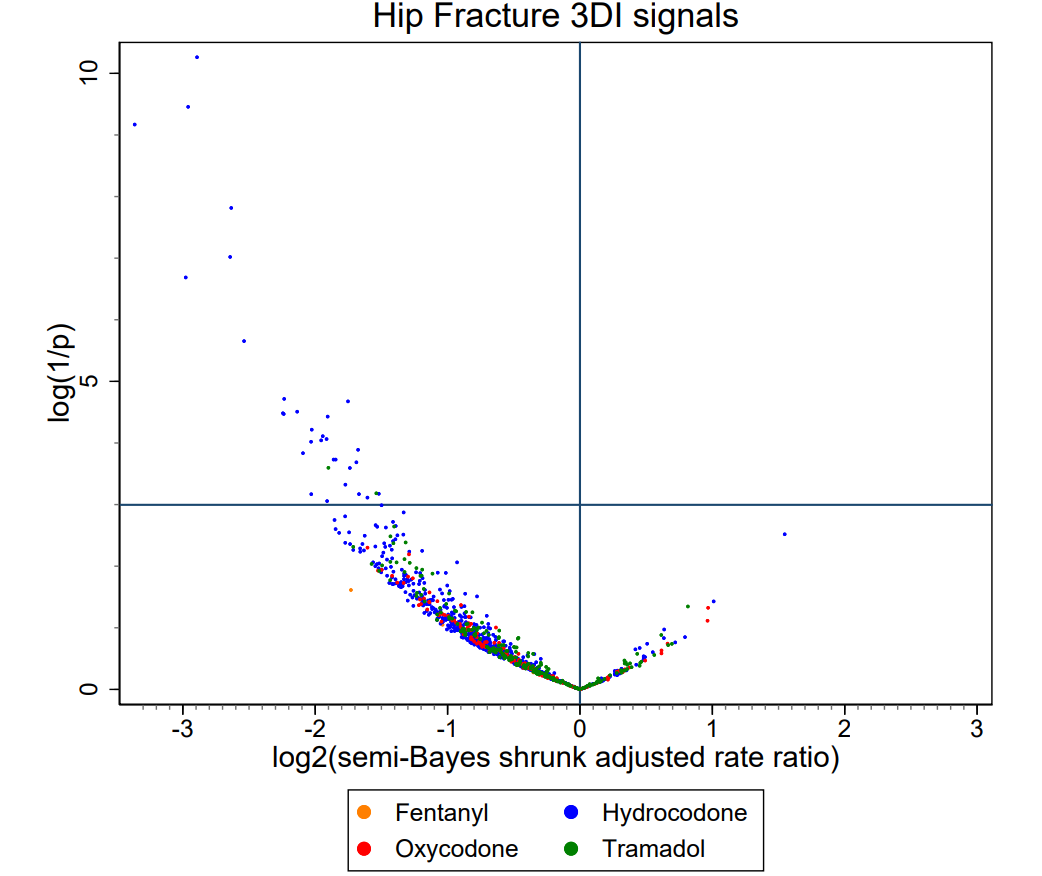
**

Semi-Bayes shrinkage prespecified a variance of 0.67, assuming that 95% of true rate ratios would fall within an unspecified 25-fold range of each other. The x-axis represents the log base 2 semi-Bayes shrunk adjusted rate ratio for opioid + precipitant base pair with candidate interacting precipitant vs. opioid + precipitant base pair. The y-axis represents the log (1 / p-value) for the semi-Bayes shrunk adjusted rate ratio. Data points in the upper right quadrant represent drug triplets with a statistically significant signal for elevated risk of typical hip fracture. We excluded signals with propoxyphene (a medical product eventually withdrawn from the United States market) from the plots, as they may have represented opioid switching rather than concomitant therapy. 3DI = drug-drug-drug interactions.
